# Supplementary material for: Mapping the spatiotemporal continuum of structural connectivity development across the human connectome in youth
Source: Nat Commun. 2026 May 15;17:6550. doi: 10.1038/s41467-026-73072-6 (PMC13381693; doi:10.1038/s41467-026-73072-6)
Supplement: Supplementary file 2 — Reporting Summary [file 41467_2026_73072_MOESM2_ESM.pdf]

nature portfolio

Corresponding author(s): Zaixu Cui

Last updated by author(s): Feb 23, 2026

Reporting Summary

Nature Portfolio wishes to improve the reproducibility of the work that we publish. This form provides structure for consistency and transparency in reporting. For further information on Nature Portfolio policies, see our Editorial Policies and the Editorial Policy Checklist.

Statistics

For all statistical analyses, confirm that the following items are present in the figure legend, table legend, main text, or Methods section.

n/a	Confirmed	
☐	☒	The exact sample size (*n*) for each experimental group/condition, given as a discrete number and unit of measurement
☐	☒	A statement on whether measurements were taken from distinct samples or whether the same sample was measured repeatedly
☐	☒	The statistical test(s) used AND whether they are one- or two-sided<br>*Only common tests should be described solely by name; describe more complex techniques in the Methods section.*
☐	☒	A description of all covariates tested
☐	☒	A description of any assumptions or corrections, such as tests of normality and adjustment for multiple comparisons
☐	☒	A full description of the statistical parameters including central tendency (e.g. means) or other basic estimates (e.g. regression coefficient) AND variation (e.g. standard deviation) or associated estimates of uncertainty (e.g. confidence intervals)
☐	☒	For null hypothesis testing, the test statistic (e.g. *F*, *t*, *r*) with confidence intervals, effect sizes, degrees of freedom and *P* value noted<br>*Give P values as exact values whenever suitable.*
☒	☐	For Bayesian analysis, information on the choice of priors and Markov chain Monte Carlo settings
☐	☒	For hierarchical and complex designs, identification of the appropriate level for tests and full reporting of outcomes
☐	☒	Estimates of effect sizes (e.g. Cohen's *d*, Pearson's *r*), indicating how they were calculated

Our web collection on statistics for biologists contains articles on many of the points above.

Software and code

Policy information about availability of computer code

Data collection	Firstly, demographic, cognitive, structural and diffusion MRI data were obtained from the NIMH Data Archive (NDA) Lifespan HCP-D release 2.0 (https://nda.nih.gov/ccf). Secondly, raw structural and diffusion MRI data were obtained from the ABCD fast-tract portal in June 2022, while the demographic, cognitive, and psychopathological measures were obtained from the ABCD release 5.1 (https://nda.nih.gov/abcd). Thirdly, neuroimage data from the devCCNP were obtained via the Science Data Bank (https://doi.org/10.57760/sciencedb.07478). Data of the EFNY and SAND studies were provided by our authors. Here, we used EFNY data collected through December 10, 2025.
Data analysis	The preprocessing of structural MRI and diffusion MRI data were conducted via FreeSurfer 7.1.1 (https://surfer.nmr.mgh.harvard.edu/) and QSIprep 0.16.0 (https://qsiprep.readthedocs.io/). Harmonization of neuroimaging data derivatives was conducted via ComBat-GAM (https://github.com/rpomponio/neuroHarmonize) and NonlinearLongitudinalComBat (https://github.com/nmd1994/NonlinearLongitudinalComBat) implementations. The Connectome Workbench (v2.1.0) was used to visualize atlases on the cortical surface.<br>Statistical analysis was conducted using R 4.1.0. R packages including mgcv 1.9-1, gamm4 0.2-6, ecostats 1.2.1, pbkrtest 0.5.5, gratia 0.10.0, lme4 2.0-1 and psych 2.4.12 were used for statistical analyses. R packages including ggplot2 4.0.2 and RColorBrewer 1.1-3 were used for visualization. All codes can be found at https://github.com/CuiLabCIBR/SCDevelopment.git.

For manuscripts utilizing custom algorithms or software that are central to the research but not yet described in published literature, software must be made available to editors and reviewers. We strongly encourage code deposition in a community repository (e.g. GitHub). See the Nature Portfolio guidelines for submitting code & software for further information.

Data

Policy information about availability of data

All manuscripts must include a data availability statement. This statement should provide the following information, where applicable:

- Accession codes, unique identifiers, or web links for publicly available datasets
- A description of any restrictions on data availability
- For clinical datasets or third party data, please ensure that the statement adheres to our policy

The HCP-Development 2.0 Release data used in this report came from DOI: 10.15154/1520708 via the NDA (https://nda.nih.gov/ccf). The ABCD 5.1 data release used in this report came from DOI: 10.15154/z563-zd24 via the NDA (https://nda.nih.gov/abcd). The fast-track data from the ABCD Study data is also available through the NDA. The devCCNP data in the Chinese Cohort is available via the Science Data Bank (https://doi.org/10.57760/sciencedb.07478). Data from the EFNY and SAND studies are available under restricted access because data collection for both datasets is ongoing. The EFNY raw data will be publicly released after completion of Phase I data collection. Source data are provided with this paper.

Research involving human participants, their data, or biological material

Policy information about studies with human participants or human data. See also policy information about sex, gender (identity/presentation), and sexual orientation and race, ethnicity and racism.

Reporting on sex and gender	Sex at birth for all the cohorts was determined by parent-report. The first dataset included 590 participants (273 males) from the HCP-D dataset used for developmental and cognitive analyses. The second dataset included 3,949 (2,075 males) participants from baseline and 3,155 (1,701 males) from 2-year follow-up of the ABCD study for developmental, cognitive and psychopathological analyses. The third dataset Chinese Cohort included 947 (481 males) scans. We did not constrain our analyses in only one sex, and we did not design any analyses to test sex differences. We included the sex at birth as a covariate in the developmental (Fig.2~6), cognitive and psychopathological analyses (Fig.7). For our primary analyses, we did not restrict the analyses to a single sex, while we tested sex differences in the age at which the alignment between SC change rates and the S-A axis crossed zero as a supplementary analysis (Fig. S8).
Reporting on race, ethnicity, or other socially relevant groupings	This study included 590 participants (344 Non-Hispanic White, 56 Non-Hispanic Black, 86 Hispanic, 43 Non-Hispanic Asian and 61 Other) from the HCP-D dataset; and 3,949 participants (2,257 Non-Hispanic White, 575 Non-Hispanic Black, 678 Hispanic, 68 Non-Hispanic Asian and 371 Other) from baseline of the ABCD study; and 3,155 participants (1,817 Non-Hispanic White, 476 Non-Hispanic Black, 525 Hispanic, 41 Non-Hispanic Asian and 296 Other) from 2-year follow-up of the ABCD study. All the scans (N=947) in the Chinese Cohort are from participants of Non-Hispanic Asian. We did not constrain our analyses in only one race, and we did not design any analyses to test race differences.
Population characteristics	This study included 590 healthy youth (273 males, aged 8-22) from the HCP-D dataset, who were recruited from 4 sites. This study also included 3,949 children (2,075 males, aged 9-10) from the baseline and 3,155 children (1,701 males, aged 11-14) from the 2-year follow-up of the ABCD study, whose neuroimaging data were all scanned by SIEMENS scanners from 13 sites. Furthermore, this study included 947 scans (481 males, aged 6-23.4) from 1,082 scans in the Chinese Cohort. The neuroimaging data of the Chinese Cohort were acquired from 3 sites.
Recruitment	See (Harms et al., 2018) for the recuitment of the HCP-D participants. See (Auchte et al. Dev Cogn Neurosci. 2018; Casey BJ, et al. Dev Cogn Neurosci. 2018) for more information of the recruitment of the ABCD participants. See (Liu et al. Dev Cogn Neurosci. 2021; Zhang et al., Eur Psychiatry. 2024) for more information of the recruitment of the Chinese Cohort participants.
Ethics oversight	The HCP-D project was approved by the institutional review boards at Washington University (IRB #201603135). Written informed consent and assent were obtained from participants over 18 years of age and parents of participants under 18 years by the WU-Minn HCP Consortium. The ABCD study was approved by the Institutional Review Board of the University of California, San Diego and other relevant institutional review boards (IRB# 160091). Before participation, parents or legal guardians provided written informed consent, and children provided verbal assent. For the Chinese Cohort, ethical approvals were obtained as follows: the devCCNP was approved by the Institutional Review Board of the Institute of Psychology, Chinese Academy of Sciences (H18017); the EFNY by the Human Research Ethics Committee of the Chinese Institute for Brain Research, Beijing; and the SAND by the Institutional Review Boards at Shandong Normal University. Written assent and informed consent were obtained from all participants and, for those under 18 years old, from their parents or legal guardians.

Note that full information on the approval of the study protocol must also be provided in the manuscript.

Field-specific reporting

Please select the one below that is the best fit for your research. If you are not sure, read the appropriate sections before making your selection.

☒ Life sciences ☐ Behavioural & social sciences ☐ Ecological, evolutionary & environmental sciences

For a reference copy of the document with all sections, see nature.com/documents/nr-reporting-summary-flat.pdf

Life sciences study design

All studies must disclose on these points even when the disclosure is negative.

Sample size	No sample size calculation was performed, as we conducted our analyses based on public datasets. We included all available data from the relevant datasets, after applying several exclusion criteria explained below.
Data exclusions	From 652 HCP-D participants, we excluded 20 participants due to incomplete diffusion magnetic resonance imaging (dMRI) data and 10 participants due to anatomical anomaly. Additionally, 18 participants under 8 years of age were excluded due to the small sample size and big head motion often reported in this age group. An additional 14 participants were excluded due to excessive head motion during dMRI scanning, identified by mean framewise displacement (FD) exceeding the mean plus three standard deviations (SD). Ultimately, we included 590 participants (273 males, aged 8.1–21.9) from the HCP-D.<br>For the ABCD study, our study exclusively utilized data from SIEMENS scanners, encompassing 5,803 scans from baseline and 4,547 scans from the 2-year follow-up, each including dMRI, associated field map, and T1-weighted imaging (T1WI). From these scans, we applied various exclusion criteria including: 1) not meeting the official imaging recommended inclusion criteria outlined in the release 4.0 notes (we adopted criteria from release 4.0 because release 5.1 was not available when the MRI processing was conducted.); 2) incomplete dMRI data or failure in unzip or format conversion process; 3) lack of parental fluency in English or Spanish; 4) lack of proficiency in English; 5) diagnosis of severe sensory, intellectual, medical or neurological issues; 6) prematurity or low birth weight (N = 2,350); 7) having contraindications to MRI scanning; 8) invalid data regarding age and sex; 9) failure in data processing; 10) excessive head motion (mean FD > Mean + 3×SD). The criteria regarding demography and healthy conditions came from a prior study. After applying these criteria, we included a total of 7,104 eligible scans for the subsequent analyses, comprising 3,949 from baseline (2,075 males, aged 8.9–11.0) and 3,155 from 2-year follow-up (1,701 males, aged 10.6–13.8).<br>As to the Chinese Cohort, we initially obtained 1,082 scans with complete dMRI and T1WI data (EFNY: N = 547; devCCNP: N = 384; SAND: N = 151). Then, we applied the following exclusion criteria: 1) severe sensory, intellectual disability (IQ under 70), medical or neurological issues; 2) prematurity or low birth weight; 3) missing or invalid age or sex information; 4) data processing failures; 5) under 6 years of age due to the small sample size; 6) excessive head motion (mean FD > Mean + 3×SD). Finally, we included 947 scans (481 males, aged 6.1–23.4) for the subsequent analyses.
Replication	First, we tested the results of developmental analyses discovered in the HCP-D dataset in the Chinese Cohort and ABCD datasets (Fig. 5). Second, we conducted several sensitivity analyses to evaluate the robustness of our findings to methodological variation including: (1) varying the number of S-A cortical systems (7 and 17 instead of 12, Fig. S10a,b); (2) reconstructing structural connectomes using the canonical Yeo-7 and Yeo-17 cortical parcellations (Fig. 6a,b); (3) regressing out Euclidean distance between system pairs when assessing S-A alignment (Fig. 6c); (4) controlling for mean whole-brain SC strength (Fig. 6d); (5) including socioeconomic status (SES; Fig. 6e) and intracranial volume (ICV; Fig. 6f) as additional covariates; (6) reconstructing connectomes from major bundle-based TractSeg25 tractography (Fig. 6g); and (7) defining an alternative S-A connectional axis based on the product of system ranks (Fig. 6h, Fig. S10c). Moreover, we tested whether the S-A connectional axis captures developmental variability when the developmental axis is defined in a data-driven manner. We derived a dominant developmental axis by applying principal component analysis (PCA) to trajectories of SC developmental rates (Fig. S11a) and compared it with three connectional axes. Furthermore, we examined the robustness of cognitive and psychopathological associations with SC strength using alternative measurements (Fig. S13).<br>Our primary results can be replicated across all the conditions listed above.
Randomization	No randomization was performed as this study does not include experimental groups.
Blinding	Blinding is not relevant to this study because it does not include experimental groups.

Reporting for specific materials, systems and methods

We require information from authors about some types of materials, experimental systems and methods used in many studies. Here, indicate whether each material, system or method listed is relevant to your study. If you are not sure if a list item applies to your research, read the appropriate section before selecting a response.

Materials & experimental systems

n/a	Involved in the study	
☒	☐	Antibodies
☒	☐	Eukaryotic cell lines
☒	☐	Palaeontology and archaeology
☒	☐	Animals and other organisms
☒	☐	Clinical data
☒	☐	Dual use research of concern
☒	☐	Plants

Methods

n/a	Involved in the study	
☒	☐	ChIP-seq
☒	☐	Flow cytometry
☐	☒	MRI-based neuroimaging

Plants

Seed stocks	*Report on the source of all seed stocks or other plant material used. If applicable, state the seed stock centre and catalogue number. If plant specimens were collected from the field, describe the collection location, date and sampling procedures.*
Novel plant genotypes	*Describe the methods by which all novel plant genotypes were produced. This includes those generated by transgenic approaches, gene editing, chemical/radiation-based mutagenesis and hybridization. For transgenic lines, describe the transformation method, the number of independent lines analyzed and the generation upon which experiments were performed. For gene-edited lines, describe the editor used, the endogenous sequence targeted for editing, the targeting guide RNA sequence (if applicable) and how the editor was applied.*
Authentication	*Describe any authentication procedures for each seed stock used or novel genotype generated. Describe any experiments used to assess the effect of a mutation and, where applicable, how potential secondary effects (e.g. second site T-DNA insertions, mosiacism, off-target gene editing) were examined.*

Magnetic resonance imaging

Experimental design

Design type	This is an observational study using diffusion MRI.
Design specifications	Number of blocks, trials or experimental units are not applicable to an observational study.
Behavioral performance measures	For HCP-D and ABCD, we used the composite score of fluid cognition from the NIH Toolbox Cognition Battery to quantify the participants' cognitive ability. The fluid cognition composite score was obtained by averaging the normalized scores from multiple cognitive tasks, including flanker inhibition, dimensional change card sort (flexibility), picture sequence memory, list sorting working memory, and pattern comparison. For children from the ABCD study, problematic behaviors were assessed using the validated parent-report version of the Child Behavior Checklist (CBCL for ages 6–18 years).

Acquisition

Imaging type(s)	diffusion MRI, structural MRI.
Field strength	3T
Sequence & imaging parameters	T1WI and dMRI data were acquired for each participant in this study. MRI data forthe HCP-D, ABCD, Chinese Cohort-EFNY, and Chinese Cohort-SAND datasets were obtained using 3T SIEMENS scanners. For the Chinese Cohort-devCCNP, MRI data were acquired using a GE Discovery MR750 3T scanner. Detailed imaging acquisition parameters are summarized in Table S4.
Area of acquisition	Whole brain.

Diffusion MRI ☒ Used ☐ Not used

Parameters

HCP-D: Two sessions of dMRI with a voxel size of 1.5 mm isotropic were acquired. The sessions used opposite phase-encoding directions to facilitate the correction of distortion induced by the Echo Planar Imaging (EPI) sequence used in dMRI scanning. Each session includes 185 diffusion directions with two b-values of 1,500 and 3,000 s/mm2, along with 14 b = 0 s/mm2 images.

ABCD: The dMRI scans were acquired at a 1.7 mm isotropic resolution comprising 7 b = 0 s/mm2 frames and 96 diffusion directions across 4 shells of b = 500 s/mm2, 1,000 s/mm2, 2,000 s/mm2 and 3,000 s/mm2. Additionally, fieldmap scans in the opposite phase-encoding direction to dMRI were acquired for EPI distortion correction.

Chinese Cohort-devCCNP: The dMRI scans were acquired at a 2 mm isotropic resolution comprising 10 b = 0 s/mm2 frames and 64 diffusion directions across 1 shell of b = 1,000 s/mm2.

Chinese Cohort-SAND: The dMRI scans were acquired at a 2 mm isotropic resolution comprising 1 b = 0 s/mm2 frames and 64 diffusion directions across 1 shell of b = 1,000 s/mm2.

Chinese Cohort-EFNY: The dMRI scans were acquired at a 1.8 mm isotropic resolution comprising 9 b = 0 s/mm2 frames and 120 diffusion directions across 4 shell of b = 500 s/mm2, 1,000 s/mm2, 2,000 s/mm2 and 3,000 s/mm2. Additionally, fieldmap scans in the opposite phase-encoding direction to dMRI were acquired for EPI distortion correction.

Preprocessing

Preprocessing software	Quality-controlled, minimally processed T1-weighted structural data from the HCP-D. This preprocessing was done using FSL 5.0.6 and FreeSurfer 5.3.0. T1-weighted structural data from the ABCD and Chinese Cohort were preprocessed using FreeSurfer 7.1.1 and the anatomical pipeline embedded in QSIPrep v0.16.0. Diffusion MRI data from HCP-D, ABCD, and Chinese Cohort were preprocessed using QSIPrep v0.16.0.
Normalization	T1-weighted image (T1WI) was spatially normalized to MNI152NLin2009cAsym through nonlinear registration. Normalization generated transformation matrices to register the atlas in MNI space to individual anatomical references. The skull-stripped T1WI in native space was used as the anatomical reference for the dMRI workflow.
Normalization template	sMRI: ICBM 152 Nonlinear Asymmetrical template version 2009c.<br>dMRI: native T1WI.

Noise and artifact removal	dMRI: Marchenko-Pastur principal component analysis (MP-PCA) denoising through MRtrix3's dwidenoise function; Gibbs unringing through MRtrix3's mrdegibbs function; B1 bias correction through MRtrix3's dwibiascorrect function; head motion, distortion and eddy current corrections through FSL's eddy tool.
Volume censoring	n/a

Statistical modeling & inference

Model type and settings	Generalized additive models (GAMs); generalized additive mixed models (GAMMs). Smooth plate regression splines served as the basic function of the smooth term, and the restricted maximal likelihood approach was used to estimate smoothing parameters.
Effect(s) tested	For each model, we evaluated the significance of the age effect by comparing the full model with a null model lacking the interested term using parametric bootstrap testing via analysis of variance for GAMs and parametric bootstrap testing via the likelihood ratio test statistic for GAMMs with 1,000 simulations.

Specify type of analysis: ☒ Whole brain ☐ ROI-based ☐ Both

Statistic type for inference (See Eklund et al. 2016)	Neither voxel or cluster-based approaches were used in the study.
Correction	The P values were adjusted using the false discovery rate (FDR) correction, with a significant threshold set at 0.05.

Models & analysis

n/a	Involved in the study
☒	☐ Functional and/or effective connectivity
☒	☐ Graph analysis
☐	☒ Multivariate modeling or predictive analysis

Multivariate modeling and predictive analysis	Developmental analyses: For each model, we set structural connectivity strength as the dependent variable, with age as a smooth term, and sex and mean FD as covariates. Per-participant random intercepts were additionally included in the GAMMs. We determined the optimal degree of freedom (k) by evaluating model fit using the AIC across k values ranging from 3 to 6. A k value of 3 was selected.<br>Cognitive analyses: We employed GAMs to assess the relationships between the structural connectivity strength and cognitive composite scores for each connection, controlling for age, sex, and mean FD.<br>Psychopathological analyses: The associations were evaluated through GAMMs while controlling age, sex, and mean FD.
