# Supplementary material for: Mapping the spatiotemporal continuum of structural connectivity development across the human connectome in youth
Source: Nat Commun. 2026 May 15;17:6550. doi: 10.1038/s41467-026-73072-6 (PMC13381693; doi:10.1038/s41467-026-73072-6)
Supplement: Supplementary file 3 — Transparent Peer Review file [file 41467_2026_73072_MOESM3_ESM.pdf]

Peer Review File

Mapping the spatiotemporal continuum of structural connectivity development across the human connectome in youth

Corresponding Author: Professor Zaixu Cui

This file contains all reviewer reports in order by version, followed by all author rebuttals in order by version.

Version 0:

Reviewer comments:

Reviewer #1

(Remarks to the Author)
This study by Xu et al. shows an impressive investigation into the spatiotemporal development of structural connectivity (SC) across childhood and adolescence. By leveraging three independent, large-scale datasets (HCP-D, ABCD, and the Chinese youth), the authors map the spatiotemporal dynamics of white matter maturation along a sensorimotor-association (S-A) axis. The paper is well-written, the logic is clear, and the figures effectively present the data.

I think that the identification of a transition period around age 15.5, where development shifts from sensorimotor strengthening to association network refinement, is a novel finding that advances our understanding of adolescent neurodevelopment. The authors also found that the S-A connectivity axis captures connectome-wide spatial variation in relationships between SC strength and both cognitive performance and psychopathology. These results support a hierarchical framework for SC development. The release of the interactive ConnectCharts is also a clear strength, as it will facilitate the reuse of these data.

Overall, I find the study rigorous and very likely to be of broad interest. Below, I have outlined several points and suggestions that would strengthen the current version of this manuscript:

The developmental turning point at ~ 15.5 yrs, in my view, is a major highlight of this study. However, given that this age range overlaps with puberty—a period known for sexual dimorphism in brain maturation. It is important to clarify if this timeline is consistent between both sexes. Did the authors test for Sex * Age interactions? Specifically, does the shift from sensorimotor to association refinement occur earlier in females? I am very interested in a breakdown of the developmental trajectories by sex and think that would add significant biological nuance to the reported timeline.

The authors define the S-A axis based on a priori biological properties. To better contextualize these findings within the broader field of network neuroscience, it would be valuable to see how this axis aligns with other established hierarchy maps. How strongly does your S-A connectional axis correlate with the principal gradient of functional connectivity (e.g., Margulies et al., 2016) or myelination maps (T1w/T2w ratios)? A comparison would help readers understand how your structural findings map onto the functional hierarchy of the cortex.

The study argues for a specific gradient of change along the S-A axis. However, during development, there is often a global increase in white matter density. To confirm that the observed effects are regionally specific and not driven by whole-brain maturational trends, can the authors try regressing out the mean whole-brain structural connectivity strength? I am curious to see whether S-A gradient effects persist when controlling for global SC fluctuations.

The manuscript links SC development to the "p-factor" of psychopathology. However, the discussion could be expanded to address the practical implications of this relationship. Please elaborate on the clinical applicability of these findings. How might clinicians eventually use these ConnectCharts to identify at-risk youth before severe symptoms emerge? A more detailed discussion of the potential clinical applications would make this section more informative for translational readers.

How do the authors hypothesize that these white matter changes couple with gray matter morphology? Does the

strengthening of sensorimotor connections precede the thinning of sensorimotor cortex?

This study relies on a group-level parcellation. Given the recent focus on "precision functional mapping", we know that network boundaries can vary significantly across individuals, particularly in higher-order association zones. I suggest the authors discuss the use of group-level atlases versus individual-level parcellations as a limitation. It is worth acknowledging that while group-level analysis is necessary for these sample size, future work uaing individual-level network mapping might reveal even stronger structure-function associations by accounting for topological variability at the individual level.

Minor points:

Page 4, lines 21-24: “We used 12 systems for the main analyses, representing an intermediate scale between the widely used 7- and 17-network frameworks, and confirmed robustness using both 7- and 17-system parcellations as well as the canonical Yeo-7 and Yeo-17 functional systems.” I think this phrasing is potentially confusing. I am not sure whether ‘7-network’ means that same standard ‘Yeo-7’ atlas or if the authors created their own subdivision of the S-A axis.

Fig. 1d. The current continuous color illustrates the S-A axis progression, but it makes it hard to see the exact spatial boundaries between adjacent systems on the cortical surface. It would be very helpful to provide a supplemental version using a discrete colomap (distnct, high-contrast color for each system). This would allow people to clearly see the specific cortical topography and boundary definitions of the 12 systems.

The capitalization of article titles is inconsistent. Please carefully standardlize the citation style throughout teh reference section.

(Remarks on code availability)
I have checked that overall the code is in a usable state and a motivated reader could reproduce the principal results of the paper

Reviewer #2

(Remarks to the Author)
This manuscript investigates the spatiotemporal development of structural connectivity across childhood and adolescence using large datasets. The authors propose that white matter maturation follows a S-A connectional axis, with early strengthening of sensorimotor connections and late adolescence strengthening of association connections. They also identified a ‘tipping point’ around age 15.5 where the dominant developmental gradient shifts. The study benefits from rigorous replication across two independent datasets, but there are a few major concerns - several conceptual and methodological aspects need clarification, and further analyses are needed before the conclusions can be fully evaluated. Detailed comments follow.

Major issues:
1. The authors derive their S-A connectional axis directly from a pre-defined cortical S-A axis and then test whether developmental patterns align with this axis. This approach is inherently confirmatory rather than discovery-driven. Given that the S-A cortical axis was itself derived from multiple neurobiological properties including developmental gradients, finding that SC development aligns with this axis is perhaps unsurprising and potentially circular. Could the authors instead test out a data-driven analysis (e.g., PCA) on the developmental trajectories themselves to identify the dominant axis of variation, then compare the explanatory power of the S-A connectional axis against competing organizational principles? If not, this should be explained as to why not, and discussed as a limitation regarding interpretation of the findings.
2. The correlation between SC-cognition effect sizes and S-A rank is rho = -0.22 with P = 0.056, which is not significant by conventional thresholds yet discussed as supporting the hypothesis. Effect sizes for individual connections should be reported. The authors should also discuss what magnitude of effect would be clinically meaningful, given that the current cognitive and psychopathological analyses reveal statistically significant but extremely small effect sizes in a massive sample (n > 7000 for ABCD).
3. Methodological rigor of results- While the results are well written they are very general descriptions of findings and in many cases, not supported by any statistics in the text, e.g. ‘ Our analysis revealed variations in age effects across all 78 connections, with the strongest effects observed in connections among primary sensorimotor systems, moderate effects in connections among higher-order association systems, and the weakest effects in connections between sensorimotor and association systems.’ Was there a statistical comparisons of effect size differences? Overall, more direct mapping of the statistical analysis to the specific results would be helpful.
4. The authors mentioned that uncorrected standard scores of cognition (without age adjustment) were used for analysis. Could the authors provide more rationale for this approach?
5. What are the considerations for including cognitive scores and p-factor into the covariates in the ComBat processing? I’m wondering how cognitive & clinical variances could impact the harmonization results. Could the authors examine the differences of including verse not including the cognitive and p-factor values and provide more detailed rationales?
6. Regarding the data harmonization with ComBat, I have a concern that this process may wash out microstructural effects in phenotypic variables that differed between sites. The authors might consider using Neuroharmonize (https://github.com/rpomponio/neuroHarmonize, Pomponio et al., 2020; NeuroImage), which is built off the ComBat algorithm but allows phenotypic differences to be preserved in the harmonization process, especially for developing age youth. Otherwise, is there a way the authors can show harmonization did not affect site differences that might be expected given phenotypic differences?
7. The p-factor approach, while increasingly popular, remains controversial. Using parent-reported symptoms in a

population-based sample with restricted range may limit validity. Can the authors conduct sensitivity analyses with alternative psychopathology operationalizations that's available with ABCD or other datasets used in this study?
8. Despite having longitudinal data from ABCD and partial longitudinal data from devCCNP, the developmental trajectory analyses largely treat data as cross-sectional. The GAMMs with random intercepts account for repeated measures but do not leverage the longitudinal structure to test key claims about within-person developmental change. Statements like "developmental trajectories vary by cognition/psychopathology level" conflate between-person and within-person effects. Can the authors analyze the latent growth curve or multilevel models to explicitly test the within-person change?
9. The "critical transition" at age 15.5 feels over-interpreted. The narrow CI of might suggest potential overfitting reflect the specific parameterization of GAM smoothing rather than a true biological discontinuity. The term "critical" implies a sensitive period with specific biological properties, which is not tested. I wonder if the longitudinal ABCD data show evidence of within-person transitions around this age.

Minor issues regarding language, typos and awkward wording:
1. P.10 Ln. 16: The alignment was "stale" before ~13 years -> should be "stable"
2. "pre-defined" "predefined" need to be standardized throughout
3. EFNY's full name needs to be consistent throughout
4. hypothesized that the 'spatiotemporal heterogeneity in SC development
would have implications in both cognitions' – change to 'hypothesized that the spatiotemporal heterogeneity in SC development would have implications *for both cognition*..'
5. Line 19 in Intro- cognition, not 'cognitions'
6. Line 19/20 – "hypothesized that large-scale SC strength declined with better
cognitive performance.." ; change to 'hypothesized that large-scale SC strength *would * decline with better cognitive performance

(Remarks on code availability)
I can confirm that detailed code is available but have not tried to implement it directly

Reviewer #3

(Remarks to the Author)
I co-reviewed this manuscript with one of the reviewers who provided the listed reports. This is part of the Nature Communications initiative to facilitate training in peer review and to provide appropriate recognition for Early Career Researchers who co-review manuscripts.

(Remarks on code availability)
Reviewed the general integrity of codes but did not perform a detailed review of specific analysis scripts. Most analyses scripts are available on the github repository with a readme file.

Version 1:

Reviewer comments:

Reviewer #1

(Remarks to the Author)
Thank you for being responsive to my comments. I have no other concerns

(Remarks on code availability)

Reviewer #2

(Remarks to the Author)
In this manuscript revision the authors made impressive good faith efforts on most points, including additional data which strengthens the results.
The core developmental finding (S-A axis organization of SC maturation) is robust across datasets, parcellations, and different harmonization approaches. There remain some minor methodological concerns about secondary analysis - for example, there still appears to be some statistical fishing on the cognition correlation, but the effect was always marginal and the authors are now appropriately hedging/tempered the conclusions. In any case, these findings don't fundamentally undermine the main contribution.

(Remarks on code availability)
Can confirm that overall the code is available and in a usable state

Reviewer #3

(Remarks to the Author)
I co-reviewed this manuscript with one of the reviewers who provided the listed reports. This is part of the Nature Communications initiative to facilitate training in peer review and to provide appropriate recognition for Early Career

Researchers who co-review manuscripts.

(Remarks on code availability)

Open Access This Peer Review File is licensed under a Creative Commons Attribution 4.0 International License, which permits use, sharing, adaptation, distribution and reproduction in any medium or format, as long as you give appropriate credit to the original author(s) and the source, provide a link to the Creative Commons license, and indicate if changes were made.
In cases where reviewers are anonymous, credit should be given to 'Anonymous Referee' and the source.
The images or other third party material in this Peer Review File are included in the article's Creative Commons license, unless indicated otherwise in a credit line to the material. If material is not included in the article's Creative Commons license and your intended use is not permitted by statutory regulation or exceeds the permitted use, you will need to obtain permission directly from the copyright holder.
To view a copy of this license, visit https://creativecommons.org/licenses/by/4.0/

We thank the editor and reviewers for their constructive feedback and thoughtful suggestions. We have thoroughly revised the manuscript and addressed all comments point by point. The corresponding revisions have been noted below each comment. Changes in the manuscript text are highlighted in gray. Notably, the validation dataset (Chinese Cohort) includes data from the ongoing EFNY project, which has enrolled additional participants since our initial analysis. We have therefore incorporated these newly available data (N = 338 additional participants) into the revised manuscript. We have also reorganized the presentation of the sensitivity analyses: cross-cohort generalizability results are now presented in **Fig. 5**, while robustness to methodological variations is presented separately in **Fig. 6**.

Reviewer #1 (Remarks to the Author):

This study by Xu et al. shows an impressive investigation into the spatiotemporal development of structural connectivity (SC) across childhood and adolescence. By leveraging three independent, large-scale datasets (HCP-D, ABCD, and the Chinese youth), the authors map the spatiotemporal dynamics of white matter maturation along a sensorimotor-association (S-A) axis. The paper is well-written, the logic is clear, and the figures effectively present the data.

I think that the identification of a transition period around age 15.5, where development shifts from sensorimotor strengthening to association network refinement, is a novel finding that advances our understanding of adolescent neurodevelopment. The authors also found that the S-A connectivity axis captures connectome-wide spatial variation in relationships between SC strength and both cognitive performance and psychopathology. These results support a hierarchical framework for SC development. The release of the interactive ConnectCharts is also a clear strength, as it will facilitate the reuse of these data.

Overall, I find the study rigorous and very likely to be of broad interest. Below, I have outlined several points and suggestions that would strengthen the current version of this manuscript:

We thank the reviewer for their positive evaluation of our study and for the constructive suggestions. We are grateful for the thoughtful feedback, and as detailed below, we have carefully addressed each point and incorporated the suggested revisions into the revised manuscript.

1. The developmental turning point at ~ 15.5 yrs, in my view, is a major highlight of this study. However, given that this age range overlaps with puberty—a period known for sexual dimorphism in brain maturation. It is important to clarify if this timeline is consistent between both sexes. Did the authors test for Sex * Age interactions? Specifically, does the shift from sensorimotor to association refinement occur earlier in females? I am very interested in a breakdown of the developmental trajectories by sex and think that would add significant biological nuance to the reported timeline.

We thank the reviewer for this insightful comment and agree that puberty-related sex differences could be relevant for interpreting the identified developmental turning point. Following the reviewer's suggestion, we conducted additional analyses to examine whether the developmental timeline differs by sex.

To assess potential sex differences in the shift timing of the alignment between structural connectivity (SC) change rates and the sensorimotor-association (S-A) connectional axis, we first fitted the developmental effects using generalized additive models for each of the 78 connections separately in females (**Fig. S8a**) and males (**Fig. S8b**). Using the same procedure as in the main analyses, we computed the age-resolved spatial alignment between SC developmental rates and the S-A connectional axis for both males and females. In both groups, alignment followed a similar developmental pattern, transitioning from negative to positive values with increasing age. Notably, the zero-crossing point, marking the shift from sensorimotor-dominant to association-dominant refinement, occurred slightly earlier in females (15.1 years; 95% CI: 14.8–15.5; **Fig. S8c**) than in males (16.2 years; 95% CI: 15.7–16.7; **Fig. S8d**). We further assessed the statistical significance of this sex difference using a participant-level permutation test. Specifically, we shuffled sex labels across all participants in the HCP-D dataset 1,000 times, and for each permutation, repeated the full estimation procedure and computed the difference in the median zero-crossing age between the two permuted groups. The observed difference in median zero-crossing age (1.1 years) was significantly larger than expected under the null distribution (P_{perm} = 0.005, **Fig. S8e**). Together, these findings suggest that the timing of the shift in SC development from sensorimotor-dominant to association-dominant is modestly advanced in females relative to males. This pattern is consistent with prior reports of earlier maturation in females for cortical structure[1] and functional connectivity[2], potentially reflecting sex differences in pubertal timing.

We have added the age-by-sex results and relevant methods to the revised manuscript:

Methods (Page 31, Para 4):

To examine potential sex differences in the timing of the developmental shift in the alignment between spatial patterns of SC developmental rates and the S-A connectional axis, we conducted sex-stratified analyses in the HCP-D dataset. GAMs were fitted separately in females (N = 317) and males (N = 273) for each of the 78 connections. Using the fitted models, we estimated the age at which the alignment between SC developmental rates and S-A axis crossed zero, following the procedure described above. We then computed the observed sex difference as the difference in the median zero-crossing age between females and males. To assess the statistical significance of this difference, we performed a participant-level permutation test. Sex labels were randomly shuffled across participants 1,000 times, and for each permutation the full sex-stratified modeling and alignment procedure was repeated to generate a null distribution of differences in median zero-crossing age. The observed sex difference in median zero-crossing age was compared with this null distribution to obtain a permutation-based *P* value.

Results (Page 10, Para 3):

Considering that this period overlaps with puberty, we further examined whether the timing of this shift differed by sex. We fitted developmental models for each of the 78 connections separately in females (N = 317; **Fig. S8a**) and males (N = 273; **Fig. S8b**), and estimated the age at which the spatial alignment between developmental rates and the S-A axis crossed zero in each group. This zero-crossing age point, marking the shift from sensorimotor-dominant to association-dominant refinement, occurred slightly earlier in females (15.1 years, 95% CI: 14.8–15.5; **Fig. S8c**) than in males (16.2 years, 95% CI: 15.7–16.7; **Fig. S8d**). Participant-level permutation testing indicated that this sex difference was statistically significant (1,000 permutations, P_{perm} = 0.005; **Fig. S8e**). Together, these findings suggest that the timing of the shift in SC development from sensorimotor-dominant to association-dominant is modestly advanced in females relative to males.

Supplementary Materials (Page 11):

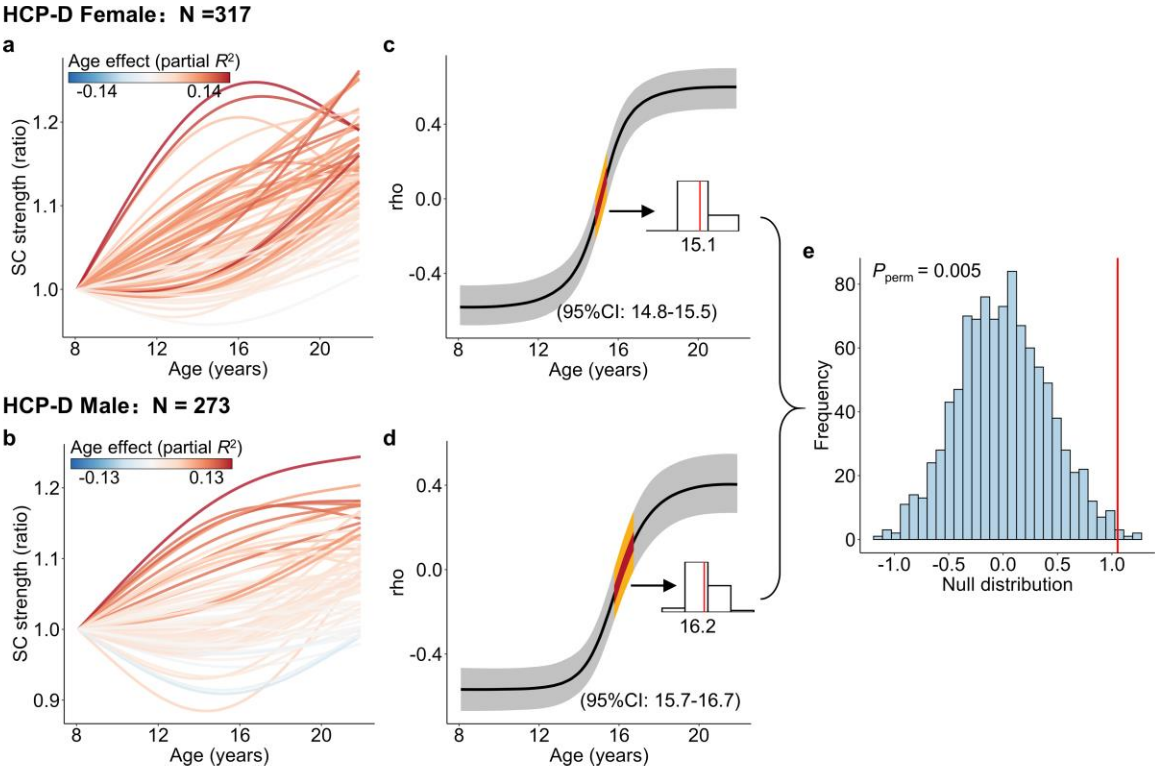

Fig. S8. Sex-stratified developmental trajectories and alignment with the S-A connectional axis. a, Developmental trajectories of SC strength in females (N = 317). Each line represents one system-to-system connection, color-coded by the magnitude of age effects (partial R^2). **b**, Developmental trajectories of SC strength in males (N = 273). **c**, Age-resolved alignment between the spatial pattern of SC developmental rates (first derivatives) and the S-A connectional axis in females, with zero alignment occurring at 15.1 years (95% CI: 14.8–15.5). **d**, Age-resolved alignment between the spatial pattern of SC developmental rates and the S-A connectional axis in males, with zero alignment occurring at 16.2 years (95% CI: 15.7–16.7). **e**, Null distribution of the difference in median zero-crossing age between females and males from 1,000 permutations of sex labels. The red vertical line represents the observed sex difference, with the corresponding permutation-based significance (P_{perm} = 0.005). SC: structural connectivity; CI: credible interval.

2. The authors define the S-A axis based on a priori biological properties. To better contextualize these findings within the broader field of network neuroscience, it would be valuable to see how this axis aligns with other established hierarchy maps. How strongly does your S-A connectional axis correlate with the principal gradient of functional connectivity (e.g., Margulies et al., 2016) or myelination maps (T1w/T2w ratios)? A comparison would help readers understand how your structural findings map onto the functional hierarchy of the cortex.

Thank you for raising this important point. The S-A cortical axis adapted from Sydnor et al. (2021)[3] was derived by integrating ten cortical maps capturing diverse neurobiological properties, including intracortical myelin[4], the principal functional gradient[5], evolutionary

expansion[6], and other neurobiological features.

At the cortical level, the S-A axis showed a strong positive correlation with the principal functional gradient[5] (Spearman's rank rho = 0.85) and a strong negative correlation with the T1w/T2w ratio[4] (rho = -0.80) across cortical vertices (**Fig. S7**). To enable direct comparison at the connectome level, we mapped these two hierarchical measures to connection-level axes using the same procedure as in the main analyses. Specifically, mean values of the functional gradient and T1w/T2w ratio were computed within each of the 12 cortical systems and converted to connection-level rankings using a sum-of-squares approach, with the resulting scores re-ranked to assign discrete integer values from 1 to 78. We found that the S-A connectional axis was highly correlated with both the functional gradient-based connectional axis (rho = 0.98) and the T1w/T2w-based connectional axis (rho = -0.90; **Fig. S7**). Together, these results demonstrate that the S-A connectional axis closely aligns with established functional and anatomical hierarchies, supporting its role as an integrative representation of hierarchical biological organization.

We have added these comparisons to the revised manuscript.

Results (Page 8, Para 1):

The S-A connectional axis was highly correlated with both the functional gradient[23]-based connectional axis (rho = 0.98; **Fig. S7**) and the T1w/T2w[24]-based connectional axis (rho = -0.90; **Fig. S7**), supporting the S-A connectional axis as an integrative representation of hierarchical cortical organization.

Supplementary Materials (Page 10):

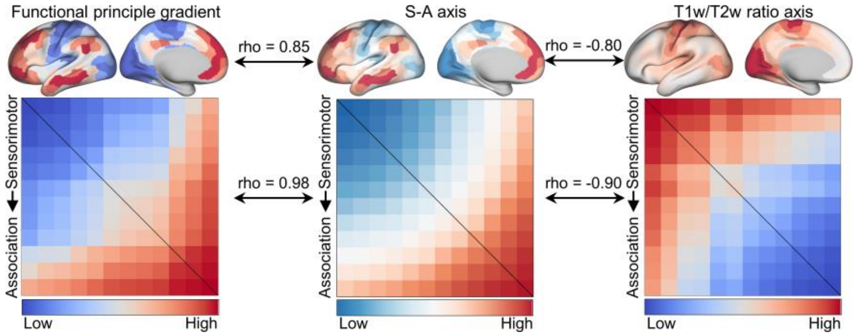

Fig. S7. Comparison of S-A axis with alternative organizational principles. Cortical maps of the S-A axis showed a strong positive correlation with the principal functional gradient (rho = 0.85) and a strong negative correlation with the T1w/T2w ratio (rho = -0.80) across cortical vertices. Mean values of the principal functional gradient and T1w/T2w ratio were computed and visualized within each of the 12 cortical systems. These system-level measures were subsequently converted to connectome-level axes using a sum-of-squares approach and rank-ordered to yield discrete integer values from 1 to 78. The S-A connectional axis was highly correlated with both the functional gradient-based connectional axis (rho = 0.98) and the T1w/T2w-based connectional axis (rho = -0.90). S-A: sensorimotor-association.

3. The study argues for a specific gradient of change along the S-A axis. However, during development, there is often a global increase in white matter density. To confirm that the observed effects are regionally specific and not driven by whole-brain maturational trends,

can the authors try regressing out the mean whole-brain structural connectivity strength? I am curious to see whether S-A gradient effects persist when controlling for global SC fluctuations.

We thank the reviewer for this important suggestion regarding potential global maturational effects. To assess whether the observed alignment with the S-A connectional axis reflects regionally specific developmental variation rather than global increases in white matter connectivity, we conducted an additional sensitivity analysis in which mean whole-brain SC strength was included as a covariate when fitting the developmental models for each connection.

After controlling for mean whole-brain SC strength, the primary findings remained highly consistent. Specifically, the second derivatives of connection-wise developmental trajectories remained strongly and positively correlated with the S-A connectional axis ranks across all connections (rho = 0.80, $P < 0.001$; **Fig. 6d** left). Moreover, the age-resolved alignment between SC developmental rates (first derivatives) and the S-A connectional axis continued to exhibit a transition from negative to positive values, with the zero-alignment point occurring at 15.4 years (95% CI: 15.2-15.6; **Fig. 6d** right), closely matching the estimate from the primary analysis.

Together, these results demonstrate that the observed S-A gradient in SC development is not driven by global changes in whole-brain connectivity strength.

We have added this analysis to the revised manuscript as a sensitivity analysis.

Supplementary Materials, Methods of sensitivity analyses (Page 2, Para 5):

The fourth sensitivity analysis assessed whether the observed developmental variations in SC persisted after controlling for global connectivity strength. To this end, we computed mean whole-brain SC strength by averaging connection strengths across all 78 system-to-system connections in the 12 × 12 large-scale connectome. Mean whole-brain SC strength was then included as an additional covariate when fitting the developmental models for each individual connection.

Results (Page 14, Para 1):

We next evaluated the robustness of our findings within the HCP-D dataset … (4) controlling for mean whole-brain SC strength (**Fig. 6d**); … Across these analyses, average second derivatives consistently correlated with S-A connectional axis ranks, and age-resolved alignment exhibited a reproducible transition from negative to positive, with zero alignment occurring near mid-adolescence (~15 years).

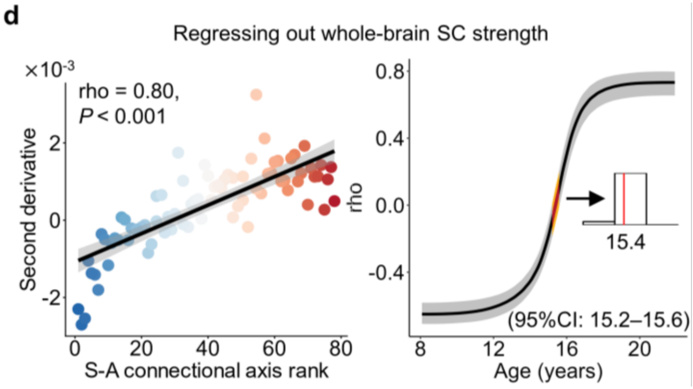

Fig. 6. Developmental variability of structural connectivity is robust to methodological variations. We validated our results using several alternative methodological approaches, including … controlling for mean whole-brain SC strength when fitting developmental models (**d**); … **a**–**h**, across all methodological replications using the HCP-D dataset, the primary findings were consistently reproduced. In each analysis, the left panel shows the correlation between the second derivatives of developmental trajectories and S-A connectional axis ranks, and the right panel shows the age-resolved alignment between developmental change rates and the S-A axis. Dots are color-coded by S-A ranks. The black line represents the median correlation value, and the gray band indicates the 95% CI. Yellow bands denote CI for the age of zero alignment, with the median and 95% CI of the transition age annotated. SC: structural connectivity; S-A: sensorimotor-association; ... CI: credible interval.

4. The manuscript links SC development to the "p-factor" of psychopathology. However, the discussion could be expanded to address the practical implications of this relationship. Please elaborate on the clinical applicability of these findings. How might clinicians eventually use these ConnectCharts to identify at-risk youth before severe symptoms emerge? A more detailed discussion of the potential clinical applications would make this section more informative for translational readers.

We thank the reviewer for this suggestion. We have expanded the Discussion to clarify the potential clinical and translational implications of the observed associations between SC development and general psychopathology.

Our findings provide a developmental reference for benchmarking SC development and characterizing deviations in clinical studies. The ConnectCharts platform operationalizes this framework by providing normative, age-associated trajectories of SC strength along the S-A connectional axis. In principle, individual SC developmental patterns could be compared against these benchmarks, especially for association connections, which showed the strongest links to psychopathology in our study.

From a translational perspective, such normative charts could eventually support risk characterization by identifying youth whose SC development deviates from typical trajectories before the emergence of clinical symptoms. Rather than relying on static case-control contrasts, this approach emphasizes trajectory-based monitoring, which may be better aligned with the dynamic nature of neurodevelopmental risk. Longitudinal tracking of SC deviations across connections ordered along the S-A axis may facilitate monitoring of symptom progression or normalization over time. Consistent with this conceptual framework, our follow-up work suggests that developmental deviations of SC strength are associated with symptom change and treatment response in youth with ADHD[7].

We have now added text to the Discussion to more explicitly articulate these potential clinical implications, while emphasizing that further validation in clinically enriched and prospective samples will be required before clinical application.

Discussion (Page 22, Para 1):

From a translational perspective, the observed relationships between SC development and general psychopathology suggest a potential role of connectome-based normative frameworks in risk characterization. The developmental trajectories characterized here provide a reference against which individual developmental patterns of SC can be compared. Deviations from typical trajectories, particularly for connections localized at the association apex of the S-A connectional axis, may index neurodevelopmental vulnerability. The ConnectCharts platform could facilitate such individualized, trajectory-based assessment and may motivate further studies examining how deviations in SC strength relate to symptom change or treatment response[48]. Importantly, clinical translation will require prospective validation in clinically enriched cohorts and careful integration with behavioral and clinical assessments.

5. How do the authors hypothesize that these white matter changes couple with gray matter morphology? Does the strengthening of sensorimotor connections precede the thinning of sensorimotor cortex?

We appreciate the reviewer's comment. Based on our findings and prior neurodevelopmental literature, we hypothesize that a coarse white matter (WM) connectome scaffold is established early in life and provides anatomical constraints on gray-matter (GM) maturation, whereas WM microstructural refinement unfolds over a more protracted period than GM morphological development.

First, converging developmental evidence indicates that long-range structural connections and key features of structural network architecture (e.g. hub configuration and hierarchical complexity) are already established in early life, with later development primarily refining connection strength and network topology[8,9]. This early WM architecture may constrain GM morphological development. Supporting this view, Liang et al. (2024)[10] reported that cortical regions connected by WM pathways show more similar developmental trajectories of cortical thickness during youth.

However, lifespan and neurodevelopmental literature suggest that WM connectivity undergoes more protracted refinement than GM morphological features. For example, cortical thickness in most regions begins to decline before age 5 (with some prefrontal and temporo-parietal areas before ~8 years)[11]; GM volume peaks between approximately 2–10 years (earlier in sensorimotor and later in frontal/temporal association regions)[12]; and cortical surface area peaks later, with sensorimotor regions before ~10 years and association regions around ~20 years[13]. In our data, SC strength of large-scale white-matter bundles among sensorimotor connections increased before age 8 and peaked around ~16 years, whereas association connections began increasing around ~13 years and continued to strengthen beyond our upper age boundary (22 years). The evidence does not clearly support a strict temporal precedence where sensorimotor WM strengthening happens first and then sensorimotor cortical thinning follows. However, the WM refinement is prolonged. This extended WM refinement may partly reflect ongoing myelination and activity-dependent myelin plasticity[14] during development.

Overall, these findings suggest that the WM scaffold established early in life constrains aspects of GM organization, while during youth both SC and GM features continue to

refine along shared developmental axes. We have added an expanded discussion reflecting this coordinated, axis-structured view of white and gray matter maturation in the revised manuscript.

Discussion (Page 20, Para 3):

This continuum mirrors prior evidence of asynchronous cortical maturation along the S-A cortical axis, including changes in gray matter morphology[7,32], intracortical myelin[11], intrinsic activity[12], and functional connectivity strength[13,33]. Together, these findings support the view that white and gray matter mature in a coordinated, axis-structured manner. An early-established white-matter scaffold[34,35] may provide anatomical constraints on cortical morphometric development[36], whereas white matter continues to undergo protracted refinement, such as ongoing myelination and activity-dependent plasticity[6], into adulthood. Our results extend this axis-structured maturation framework to the structural connectome and provide initial evidence that the developmental sequence of SC conforms to the S-A axis across the human connectome.

6. This study relies on a group-level parcellation. Given the recent focus on "precision functional mapping", we know that network boundaries can vary significantly across individuals, particularly in higher-order association zones. I suggest the authors discuss the use of group-level atlases versus individual-level parcellations as a limitation. It is worth acknowledging that while group-level analysis is necessary for these sample size, future work using individual-level network mapping might reveal even stronger structure-function associations by accounting for topological variability at the individual level.

We thank the reviewer for this valuable suggestion. We agree that recent precision functional mapping studies demonstrate substantial inter-individual variability in network boundaries, especially within higher-order association cortex[15-19]. We have added text to the Discussion noting this as a limitation and emphasizing that future work combining individualized parcellations with SC reconstruction may better accommodate topological variability and potentially reveal stronger structure-function associations.

Discussion (Page 22, Para 2):

Second, recent advances in precision functional mapping highlight pronounced inter-individual variability in functional network boundaries, particularly within higher-order association cortex[53-57]. Future studies employing individual-level parcellations to define personalized SC may improve the interpretability of structure-function relationships by accounting for this topological variability.

Minor points:

1. Page 4, lines 21-24: “We used 12 systems for the main analyses, representing an intermediate scale between the widely used 7- and 17-network frameworks, and confirmed robustness using both 7- and 17-system parcellations as well as the canonical Yeo-7 and Yeo-17 functional systems.” I think this phrasing is potentially confusing. I am not sure whether ‘7-network’ means that same standard ‘Yeo-7’ atlas or if the authors created their own subdivision of the S-A axis.

We apologize for the ambiguity. Here, we conducted two separate robustness analyses: (i) S-A axis-based parcellations created by equally partitioning the cortex into 7 or 17 bins along the S-A axis, and (ii) the canonical Yeo-7 and Yeo-17 functional network

parcellations. We have revised the text to clearly distinguish these two parcellation schemes.

Results (Page 4, Para 2):

We used 12 systems for the main analyses, representing an intermediate scale relative to the widely used Yeo-7 and Yeo-17 functional network parcellations. Robustness was assessed using alternative parcellations in which the cortex was subdivided into (i) 7 or 17 systems based on S-A axis ranks, and (ii) the canonical Yeo-7 and Yeo-17 functional network parcellations[19].

2. Fig. 1d. The current continuous color illustrates the S-A axis progression, but it makes it hard to see the exact spatial boundaries between adjacent systems on the cortical surface. It would be very helpful to provide a supplemental version using a discrete colormap (distinct, high-contrast color for each system). This would allow people to clearly see the specific cortical topography and boundary definitions of the 12 systems.

We appreciate this suggestion. We have added a supplemental figure showing the 12 S-A axis-based systems using a discrete, high-contrast colormap to clearly delineate system boundaries (**Fig. S4**).

Results (Page 4, Para 2):

These systems progressively spanned from primary sensorimotor to higher-order association cortices (**Fig. 1e**, **Fig. S4**), with each cortical system comprising regions with similar neurobiological profiles in anatomy, function, evolutionary expansion, metabolism, and gene expression.

Supplementary Materials (Page 7):

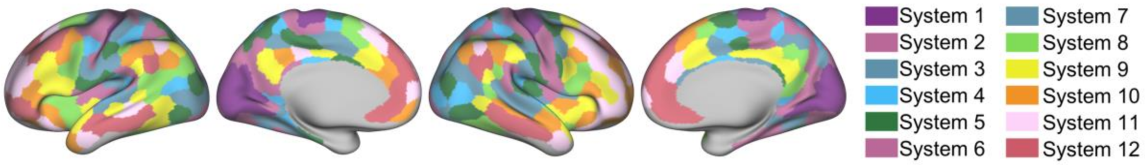

Fig. S4. Discrete visualization of the 12 cortical systems defined by partitioning the S-A axis.

3. The capitalization of article titles is inconsistent. Please carefully standardize the citation style throughout the reference section.

We apologize for this oversight. We have standardized the capitalization of article titles and corrected the formatting throughout the reference section. Now, we have standardized the reference titles so that each word is capitalized (title case) and updated the reference list.

Methods (Page 28, Subtitle):

STATISTICAL ANALYSIS → Statistical analysis

Supplemental information (Page 36, Subtitle), Supplementary Materials (Page 2, Subtitle):

Supplementary Text → Supplementary text

References (Page 37–41):

10 Sydnor, V. J. *et al.* Neurodevelopment of the Association Cortices: Patterns, Mechanisms, and Implications for Psychopathology. *Neuron* **109**, 2820-2846 (2021). https://doi.org/10.1016/j.neuron.2021.06.016

12 Sydnor, V. J. *et al.* Intrinsic Activity Development Unfolds Along a Sensorimotor-Association Cortical Axis in Youth. *Nat Neurosci* 26, 638-649 (2023). https://doi.org/10.1038/s41593-023-01282-y

16 Le Bihan, D. Looking into the Functional Architecture of the Brain with Diffusion MRI. *Nat. Rev. Neurosci.* **4**, 469-480 (2003). https://doi.org/10.1038/nrn1119

21 Smith, R. E., Tournier, J.-D., Calamante, F. & Connelly, A. Anatomically-Constrained Tractography: Improved Diffusion MRI Streamlines Tractography through Effective Use of Anatomical Information. *Neuroimage* **62**, 1924-1938 (2012). https://doi.org/10.1016/j.neuroimage.2012.06.005

26 Weintraub, S. *et al.* Cognition Assessment Using the NIH Toolbox. *Neurology* **80**, S54-S64 (2013). https://doi.org/10.1212/WNL.0b013e3182872ded

44 Association, A. P. Diagnostic and Statistical Manual of Mental Disorders. (American psychiatric association, 2013).

59 Liu, S., Abdellaoui, A., Verweij, K. J. H. & van Wingen, G. A. Replicable Brain-Phenotype Associations Require Large-Scale Neuroimaging Data. *Nat Hum Behav* 7, 1344-1356 (2023). https://doi.org/10.1038/s41562-023-01642-5

Reviewer #1 (Remarks on code availability):

I have checked that overall the code is in a usable state and a motivated reader could reproduce the principal results of the paper

We appreciate your careful inspection.

Reviewer #2 (Remarks to the Author):

This manuscript investigates the spatiotemporal development of structural connectivity across childhood and adolescence using large datasets. The authors propose that white matter maturation follows a S-A connectional axis, with early strengthening of sensorimotor connections and late adolescence strengthening of association connections. They also identified a 'tipping point' around age 15.5 where the dominant developmental gradient shifts. The study benefits from rigorous replication across two independent datasets, but there are a few major concerns - several conceptual and methodological aspects need clarification, and further analyses are needed before the conclusions can be fully evaluated. Detailed comments follow.

We thank the reviewer for the summary of our work and for recognizing the strengths of the cross-dataset replication. We appreciate the constructive feedback and have carefully addressed the conceptual and methodological concerns raised. We note that one of the independent validation datasets (Chinese Cohort) includes data from an ongoing EFNY project, which has enrolled additional participants since our initial analysis. We have therefore incorporated these newly available participants (N=338 additional participants) into the revised manuscript and updated the cross-dataset generalizability analyses, now presented in an independent **Fig. 5**. As detailed below, we have addressed all comments and incorporated the suggested revisions into the revised manuscript.

Major issues:

1. The authors derive their S-A connectional axis directly from a pre-defined cortical S-A axis and then test whether developmental patterns align with this axis. This approach is inherently confirmatory rather than discovery-driven. Given that the S-A cortical axis was itself derived from multiple neurobiological properties including developmental gradients, finding that SC development aligns with this axis is perhaps unsurprising and potentially circular. Could the authors instead test out a data-driven analysis (e.g., PCA) on the developmental trajectories themselves to identify the dominant axis of variation, then compare the explanatory power of the S-A connectional axis against competing organizational principles? If not, this should be explained as to why not, and discussed as a limitation regarding interpretation of the findings.

We thank the reviewer for this thoughtful comment and are pleased to clarify this issue. The sensorimotor-association (S-A) axis proposed by Sydnor et al. (2021)[3] was derived by integrating ten representative hierarchical features, such as cortical myelination and the principal gradient of functional connectivity (**Response Table 1**). Although several studies have shown that the S-A axis captures variability in cortical developmental patterns, such as intrinsic activity[20] and functional connectivity strength[21], the definition of the S-A axis itself did not explicitly incorporate any developmental gradients.

Response Table 1. Hierarchical features underlying the derivation of the S-A axis (adapted from Sydnor et al., 2021[3])

Neurobiological cortical maps	Quantification methods
Anatomical hierarchy	T1w/T2w ratio to index cortical myelination.
Functional hierarchy	Principal gradient of functional connectivity.
Evolutionary hierarchy	Macaque-to-human cortical expansion.

Allometric scaling	Relative extent of areal scaling with scaling of overall brain size.
Aerobic glycolysis	Positron emission tomography measures of oxygen consumption and glucose use.
Cerebral blood flow	Arterial spin labeling.
Gene expression	The first principal component of brain-expressed genes.
NeuroSynth	The first principal component of NeuroSynth meta-analytic decoding.
Externopyramidization	The ratio of supragranular pyramidal neuron soma size to infragranular pyramidal neuron soma size.
Cortical thickness	Structural MRI.

Nevertheless, we agree that it is important to compare the explanatory power of the S-A connectional axis with alternative, data-driven organizational principles. To this end, we first sought to extract a data-driven dominant axis of developmental variation directly from the trajectories. Because our goal was to characterize the spatial organization of developmental change across connections, we focused on age-resolved change rates or slopes rather than age-specific structural connectivity (SC) magnitude. We defined the data-driven dominant developmental axis as PC1 loadings of the SC change-rate trajectories (**Fig. S11a**).

We compared this dominant developmental axis with three competing organizational principles: the S-A axis[3], the functional principal gradient axis[5], and the anatomical myelination-related axis indexed by the T1-weighted/T2-weighted (T1w/T2w) ratio[4]. For the latter two measures, we computed the mean functional gradient and mean T1w/T2w ratio within each of the 12 systems and converted these system-level measures into connection-level axes using a sum-of-squares approach, assigning discrete integer ranking from 1 to 78, analogous to the definition of the S-A connectional axis. Spearman's rank correlation analyses revealed that the S-A connectional axis showed the strongest association with the dominant developmental axis (rho = 0.80, $P < 0.001$; **Fig. S11b**), followed by the functional gradient axis (rho = 0.76, $P < 0.001$; **Fig. S11c**) and the T1w/T2w ratio axis (rho = −0.57, $P < 0.001$; **Fig. S11d**). Together, these results indicate that the S-A axis explains the dominant, data-driven pattern of SC developmental variation more strongly than alternative organizational principles, supporting its relevance beyond a purely confirmatory framework.

We have added this sensitivity analysis in the revised manuscript.

Supplementary Materials, Methods of sensitivity analyses (Page 3, Para 4):

Finally, to derive a data-driven dominant axis of SC developmental variation, we applied principal component analysis (PCA) to connection-wise trajectories of SC developmental rates (**Fig. 4a**; **Fig. S11a**). Specifically, we assembled the change-rate trajectories into a matrix with connections as rows and age points as columns, and performed PCA on this matrix. The loadings of the first principal component (PC1) across connections were taken as the dominant, data-driven developmental axis. We then assessed the spatial correspondence between this developmental axis and the S-A connectional axis, as well as alternative connectional axes derived from principal functional gradient and T1w/T2w measures, using Spearman's rank correlation.

Results (Page 14, Para 1):

Moreover, we tested whether the S-A connectional axis captures developmental variability when the developmental axis is defined in a data-driven manner. We derived a dominant developmental axis by applying principal component analysis (PCA) to trajectories of SC developmental rates (**Fig. S11a**) and compared it with three connectional axes. The S-A connectional axis best explained the data-driven axis, showing a stronger correspondence (rho = 0.80, *P* < 0.001; **Fig. S11b**) than alternative organization principles, including the functional gradient[23]-based connectional axis (rho = 0.76, *P* < 0.001; **Fig. S11c**) and the T1w/T2w[24]-based connectional axis (rho = -0.57, *P* < 0.001; **Fig. S11d**).

Supplementary Materials (Page 14):

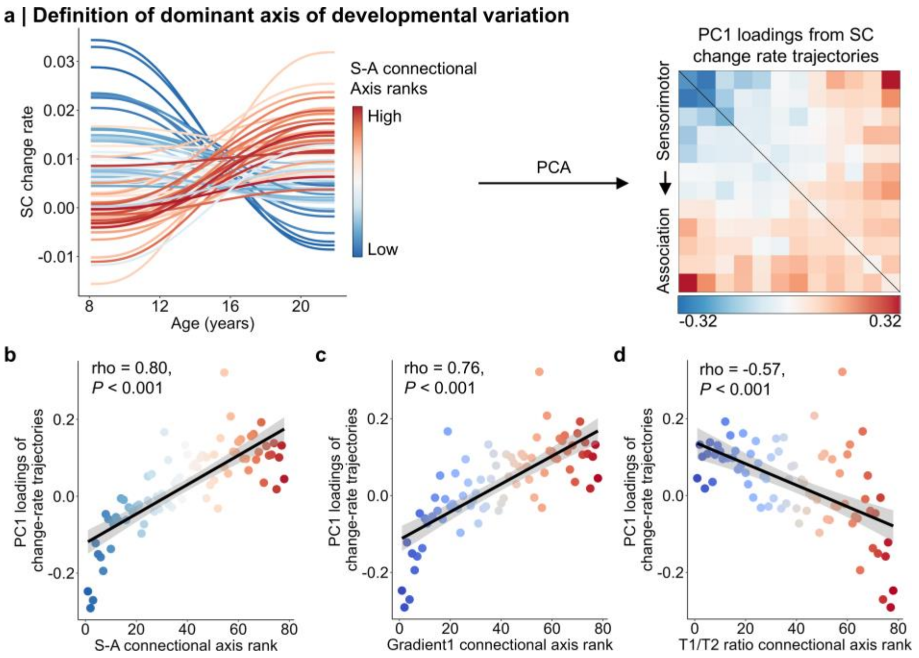

Fig. S11. Data-driven developmental axis and its alignment with connectional axes. a, The data-driven dominant developmental axis was defined as the loadings of the first principal component (PC1) obtained from PCA applied to SC change-rate (first-derivative) trajectories. **b**, The data-driven developmental axis was strongly correlated with the S-A connectional axis across all connections (rho = 0.80, *P* < 0.001). **c**, The data-driven developmental axis was strongly associated with the connectional axis derived from the principal functional gradient (rho = 0.76, *P* < 0.001). **d**, The data-driven developmental axis was negatively correlated with the T1w/T2w-based connectional axis (rho = -0.57, *P* < 0.001). SC: structural connectivity; PCA: principal component analysis; PC1: the first principal component; S-A: sensorimotor-association.

2. The correlation between SC-cognition effect sizes and S-A rank is rho = -0.22 with P = 0.056, which is not significant by conventional thresholds yet discussed as supporting the hypothesis. Effect sizes for individual connections should be reported. The authors should also discuss what magnitude of effect would be clinically meaningful, given that the current cognitive and psychopathological analyses reveal statistically significant but extremely small effect sizes in a massive sample (n > 7000 for ABCD).

We thank the reviewer for this thoughtful comment and agree that careful interpretation of effect sizes is essential, particularly in large samples. As the reviewer correctly noted, the correlation between connection-wise effect sizes and S-A connectional axis ranks was rho = -0.22 (P = 0.056), which did not meet the conventional two-sided threshold of $P < 0.05$ and therefore should be described as a trend-level correlation. The S-A alignment was tested across 78 connections, and this sample size may limit statistical power. We have revised the text to describe the finding as trend-level correlation.

Results (Page 16, Para 2):

Original text:

A connectome-wide analysis confirmed a trend toward more negative effects at higher S-A ranks (rho = -0.22, P = 0.056; **Fig. 6c**), suggesting that SC-cognition associations varied spatially along the S-A continuum.

Revised text:

A connectome-wide analysis confirmed a trend toward more negative effects at higher S-A ranks (rho = -0.22, P = 0.056; **Fig. 7c**), suggesting a trend-level correlation between SC–cognition associations and the S-A axis ranks.

Discussion (Page 21, Para 3):

Original text:

Our findings also revealed that SC strength was associated with higher-order cognitive performance, with the S-A connectional axis capturing the spatial variation in these associations.

Revised text:

Our findings also revealed that SC strength was associated with higher-order cognitive performance, and that SC–cognition associations showed a trend toward systematic variation along the S-A connectional axis.

To further assess the robustness of this trend-level alignment, we additionally performed a permutation test by randomly permuting the SC–cognition association effect sizes (T values) across connections 1,000 times and recomputing the Spearman's correlation with S-A ranks to generate a null distribution. The observed correlation was more extreme than expected under the null (P_{perm} = 0.030), providing additional support that SC–cognition associations tend to align with the S-A connectional axis.

Following the reviewer's suggestion, we now also explicitly report partial correlation coefficients as effect sizes for SC–cognition and SC–*p*-factor associations (**Fig. S12a**,**b** left) and visualize their distributions (**Fig. S12a**,**b** right). Across the 78 connections, SC–cognition associations ranged from r = -0.11 to -0.01, while SC–*p*-factor associations ranged from r = -0.03 to 0.05. Although small in magnitude, these effect sizes fall within the range typically observed in large-scale brain–behavior association studies[22-24]. For example, Marek et al. (2022, Nature)[22] reported that the highly reproducible brain-wide associations exhibited $|r|$ values below 0.16 for cognition and below 0.05 for psychopathology, supporting the reliability of the SC-behavior associations we observed.

We have revised the manuscript to report effect sizes for individual connections.

Results (Page 16, Para 2; Page 17, Para 2):

In the ABCD dataset, 73 of 78 connections showed significant associations between SC strength and cognition ($P_{FDR} < 0.05$; **Fig. 7b**; partial correlation coefficients in **Fig. S12a**).

Eleven connections showed significant positive associations ($P_{FDR} < 0.05$), indicating greater SC strength with a higher *p*-factor score (**Fig. 7f**; partial correlation coefficients in **Fig. S12b**).

Supplementary Materials (Page 14):

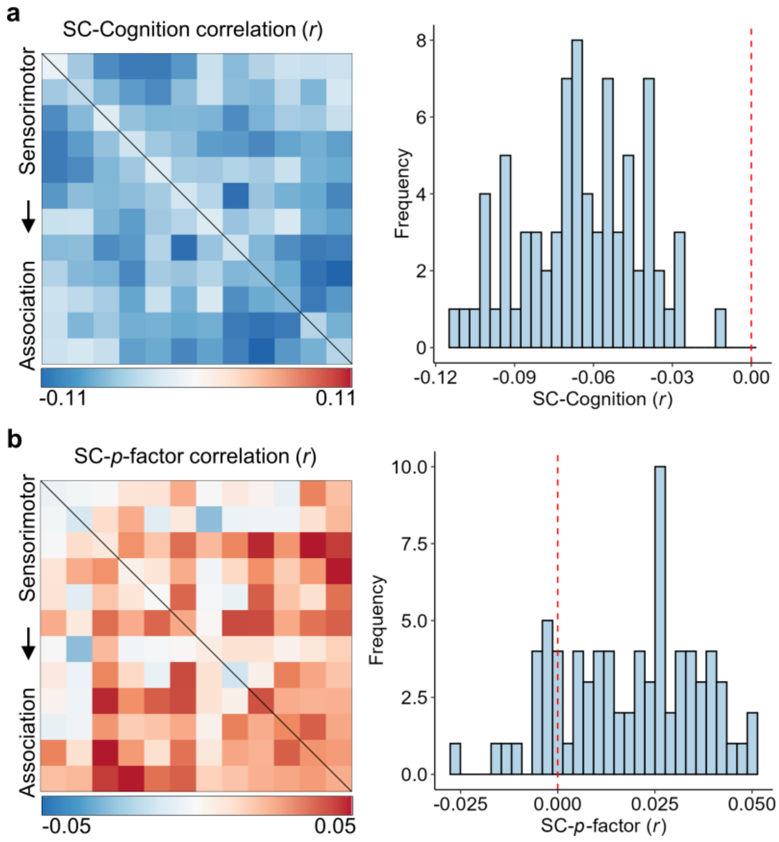

Fig. S12. Partial correlation coefficients of SC–cognition and SC–*p*-factor associations. a, Partial correlation coefficients between SC strength and fluid composite cognition scores, controlling for age, sex, and head motion. Correlation coefficients ranged from -0.11 to -0.01. **b**, Partial correlation coefficients between SC strength and *p*-factor scores, controlling for age, sex, and head motion. Correlation coefficients ranged from -0.03 to 0.05. SC: structural connectivity.

We agree that the magnitude of effects required for clinical relevance is an important consideration. In large population-based neuroimaging studies, brain-behavior associations are typically small, and few consensus thresholds define what constitutes a clinically meaningful effect at the level of individual connections. The SC-behavior correlations observed here are modest and would not support direct clinical translation. We now explicitly acknowledge in the Discussion that the modest effect sizes limit direct

clinical translation and that further work in clinically enriched and longitudinal samples will be necessary to evaluate translational potential.

Discussion (Page 22, Para 3):

Fourth, although statistically significant, the effect sizes observed for both cognitive and psychopathological effects on SC were relatively small. Prior work has consistently demonstrated that effect sizes tend to be inflated in small samples, whereas larger samples provide more accurate estimates of the true effect size[58-60]. Nevertheless, the modest magnitude of the present SC-behavior associations limits their interpretability and precludes direct clinical translation. While the observed spatial organization of developmental effects across the connectome is robust at the group level, determining whether such patterns translate into clinically meaningful prediction or risk stratification will require prospective validation in clinically enriched and longitudinal samples.

3. Methodological rigor of results- While the results are well written they are very general descriptions of findings and in many cases, not supported by any statistics in the text, e.g. 'Our analysis revealed variations in age effects across all 78 connections, with the strongest effects observed in connections among primary sensorimotor systems, moderate effects in connections among higher-order association systems, and the weakest effects in connections between sensorimotor and association systems.' Was there a statistical comparisons of effect size differences? Overall, more direct mapping of the statistical analysis to the specific results would be helpful.

We thank the reviewer for this constructive comment. We have carefully revised the manuscript and incorporated additional statistical results to support the descriptive statements presented in the Results section.

First, for the example raised by the reviewer, we conducted formal comparisons of age effect sizes (partial R^2) among the three connection types: sensorimotor–sensorimotor (SS), sensorimotor–association (SA), and association–association (AA). For each pair of connection types, we computed Cohen's d to quantify the difference in partial R^2. We then performed a permutation test in which connection-type labels were randomly shuffled for 1,000 times and Cohen's d was recomputed for each pair of types to generate a null distribution. Permutation P values were defined as the proportion of permuted Cohen's d values that were at least as extreme as the observed Cohen's d. Permutation testing (1,000 permutations) revealed that SS connections exhibited the strongest age effects, followed by AA connections and then SA connections (SS vs. SA, Cohen's d=-1.91, $P_{perm} < 0.001$; SS vs. AA, Cohen's d=-1.24, $P_{perm} = 0.002$; SA vs. AA, Cohen's d=0.75, $P_{perm} < 0.001$, **Fig. S5a**).

Second, we conducted statistical analyses to support the statement that "second derivatives displayed substantial heterogeneity across the connectome, with positive values predominantly in association–association connections and negative values in sensorimotor–sensorimotor connections (**Fig. 2c**)." For each type of connections, we computed Cohen's d to quantify the difference between second derivatives and zero. Specifically, permutation tests showed that second-derivative values for SS connections were significantly lower than zero (Cohen's d = -1.76, $P_{perm} < 0.001$, **Fig. S5b**), whereas second-derivative values for AA connections were significantly greater than zero (Cohen's d = 0.96, $P_{perm} < 0.001$, **Fig. S5b**).

We have added statistical mapping in the revised manuscript.

Methods (Page 30, Para 3):

To compare developmental statistics across connection types, we classified the 12 systems into sensorimotor ("S") and association ("A") groups based on their overlap with the Yeo-7 networks: the four systems closest to the sensorimotor pole (most overlapping with visual and somatomotor networks) were assigned to S, and the remaining eight systems to A. The 78 connections were then categorized as SS, SA, or AA. We used Cohen's d to quantify (i) differences in age effects between pairs of connection types and (ii) whether the second derivatives within each connection type differed from zero. Statistical significance was assessed using permutation testing by shuffling connection-type labels 1,000 times to generate a null distribution of Cohen's d. Permutation *P* values were defined as the proportion of permuted Cohen's d values at least as extreme as the observed value.

Results (Page 6, Para 1,2):

Our analysis revealed variations in age effects across all 78 connections, with the strongest effects observed in connections among primary sensorimotor systems (SS), moderate effects in connections among higher-order association systems (AA), and the weakest effects in connections between sensorimotor and association systems (SA, SS > AA > SA, all pairwise permutation tests, $P_{perm} < 0.05$, **Fig. S5a**).

We observed that the second derivatives displayed a substantial heterogeneity across the connectome, with positive values predominantly in AA connections and negative values in SS connections (SS < 0, AA > 0, $P_{perm} < 0.001$, **Fig. S5b**).

Supplementary Materials (Page 8):

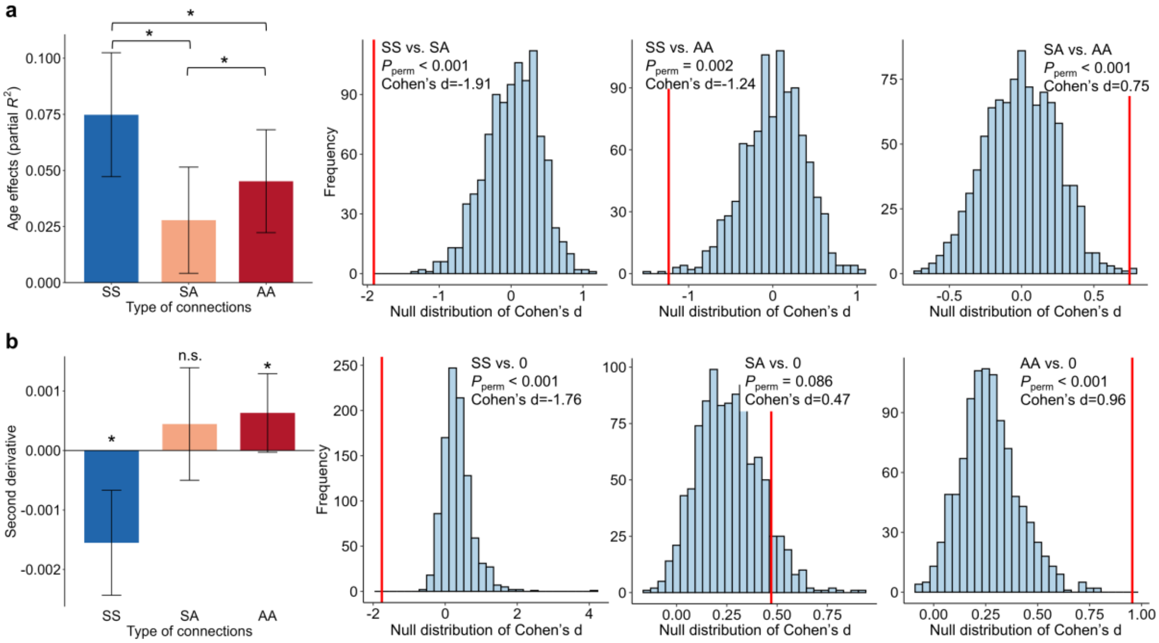

Fig. S5. Comparison of developmental features across different types of connections. a, The 12 systems were classified into two categories based on their spatial overlap with the Yeo-7 networks. The four systems closest to the sensorimotor pole were assigned to the sensorimotor

group ("S"), and the remaining eight systems were assigned to the association group ("A"). Accordingly, 78 connections were categorized into three types, SS, SA, and AA. The mean age effects (partial R^2) and corresponding standard deviations are shown for each connection type. Pairwise differences were quantified using Cohen's d. Null distributions were generated by permutation testing (1,000 permutations; light blue), and observed Cohen's d values are shown as red vertical lines. All pairwise differences were significant. **b**, Mean second derivatives and corresponding standard deviations are shown for each connection type, and differences between each connection type and zero were quantified using Cohen's d. Permutation tests (1,000 permutations) indicated that second-derivative values for SS connections were significantly lower than zero, whereas those for AA connections were significantly greater than zero. SS, sensorimotor-sensorimotor; SA, sensorimotor-association; AA, association-association; *: $P_{perm} < 0.05$; n.s.: not significant.

4. The authors mentioned that uncorrected standard scores of cognition (without age adjustment) were used for analysis. Could the authors provide more rationale for this approach?

We thank the reviewer for raising this important point. In the original analyses, we selected the uncorrected NIH Toolbox Fluid Cognition standard scores following prior youth cognition studies[25,26], and we additionally controlled for age when assessing the SC-cognition associations.

To address the reviewer's concern, we conducted a sensitivity analysis using age-corrected standard scores for the NIH Toolbox Fluid Cognition composite to confirm that our findings were robust to the choice of score standardization. In this analysis, associations between SC strength and cognitive scores were tested while controlling for sex and head motion; age was not included as a covariate to avoid double adjustment[27]. The results were highly consistent with the primary analyses: the corresponding association effect sizes correlated significantly with the S-A connectional axis ranks (rho = -0.32, *P* = 0.004, **Fig. S13a**), and the developmental trajectories exhibited different patterns across varied cognitive performance (**Fig. S13b**). We have added this sensitivity analysis and the corresponding results to the revised manuscript:

Methods (Page 26, Para 1; Page 32, Para 3):

Notably, uncorrected standard scores (without age adjustment) were used in the primary analyses, and age-corrected standard scores were used in a sensitivity analysis to confirm that our findings were not dependent on the score normalization approach.

For the sensitivity analysis using age-corrected cognitive scores, the smooth term of age was removed to avoid double adjustment[102].

Results (Page 16, Para 2):

The spatial alignment of SC–cognition associations with the S-A connectional axis remained robust, and the trajectory patterns across cognitive levels remained consistent when using age-corrected fluid composite scores (**Fig. S13a,b**).

Supplementary Materials (Page 16):

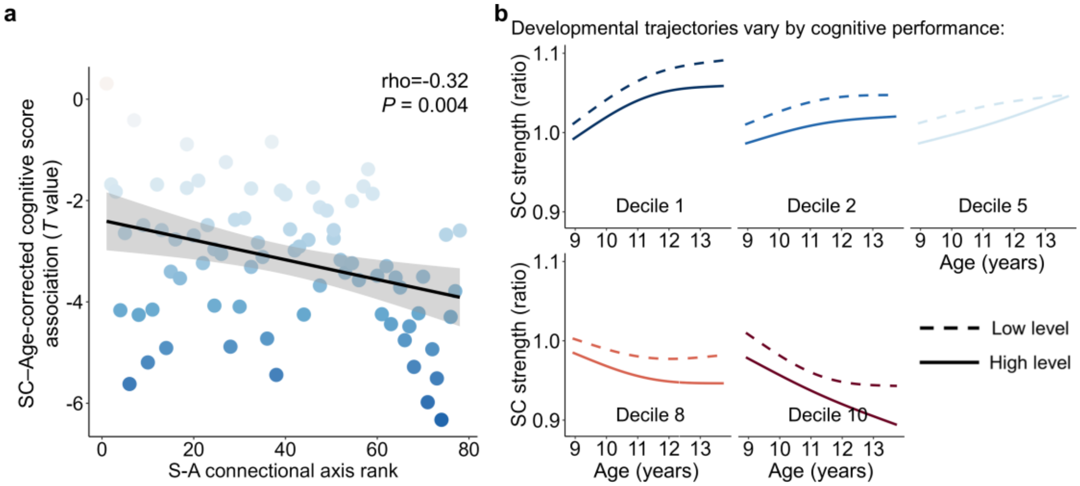

Fig. S13. Sensitivity analyses using alternative measures of cognition and psychopathology. a, Across all connections, the effect sizes of SC–cognition associations were significantly correlated with S-A connectional axis ranks (rho = -0.32, P = 0.004) when using age-corrected NIH Toolbox fluid composite scores. **b**, Developmental trajectories of SC strength are shown for groups with low (10th percentile) and high (90th percentile) baseline cognitive performance across five deciles of the S-A connectional axis.

5. What are the considerations for including cognitive scores and p-factor into the covariates in the ComBat processing? I'm wondering how cognitive & clinical variances could impact the harmonization results. Could the authors examine the differences of including versus not including the cognitive and p-factor values and provide more detailed rationales?

We are pleased to clarify. In ComBat (ComBat-GAM and NonlinearLongitudinalComBat), the model can be written conceptually as:

$$Y_{ij} = biological\ effects_{ij} + site\ effects_j + \epsilon_{ij}$$

Biological effects are defined by the covariates included in the model (e.g., age, sex, head motion, cognition, and *p*-factor), which are represented in the design matrix. Thus, the primary purpose of including cognitive scores and the *p*-factor as covariates in the ComBat procedure is to preserve variance associated with cognition and psychopathology in the brain measure of interest, preventing these biologically meaningful effects from being inadvertently absorbed into site-related batch effects during harmonization[28,29]. This harmonization strategy was applied specifically for cognitive and psychopathological analyses. For developmental analyses, only age, sex, and head motion were included as covariates. This approach is consistent with prior studies, which tailored the ComBat covariate set to the variables of interest[30-32]. We have clarified the detailed rationale in the revised manuscript.

Methods (Page 34, Para 2):

For analyses involving cognitive and psychopathological measures, the corresponding cognitive and psychopathological variables were additionally included as covariates to ensure that these phenotypic effects were preserved during harmonization, following established practice in prior studies[104-106].

To evaluate whether including cognition or the *p*-factor as ComBat covariates influenced SC–cognition or SC–*p*-factor associations, we directly compared association effect sizes (*T* values) derived from data harmonized with versus without the corresponding behavioral covariate. Both the resulting SC–cognition and SC–*p*-factor effect-size maps were highly similar between the two harmonization approaches, showing only minimal differences and a near-perfect correspondence (Pearson's $r \approx 1$; **Response Fig. 1a,b**). Importantly, the alignment between the spatial pattern of SC–behavior associations and the S-A connectional axis was also preserved when harmonization was performed without behavioral covariates (cognition: rho = -0.22, $P = 0.056$; *p*-factor: rho = 0.50, $P < 0.001$; **Response Fig. 1c,d**). In addition, we observed similar decile-level developmental trajectory patterns across cognition and *p*-factor levels under both harmonization choices (**Response Fig. 1e,f**). Together, these results indicate that harmonization covariate selection has a negligible impact on connection-specific effect sizes or the spatial organization of SC–cognition and SC–*p*-factor associations in our analyses.

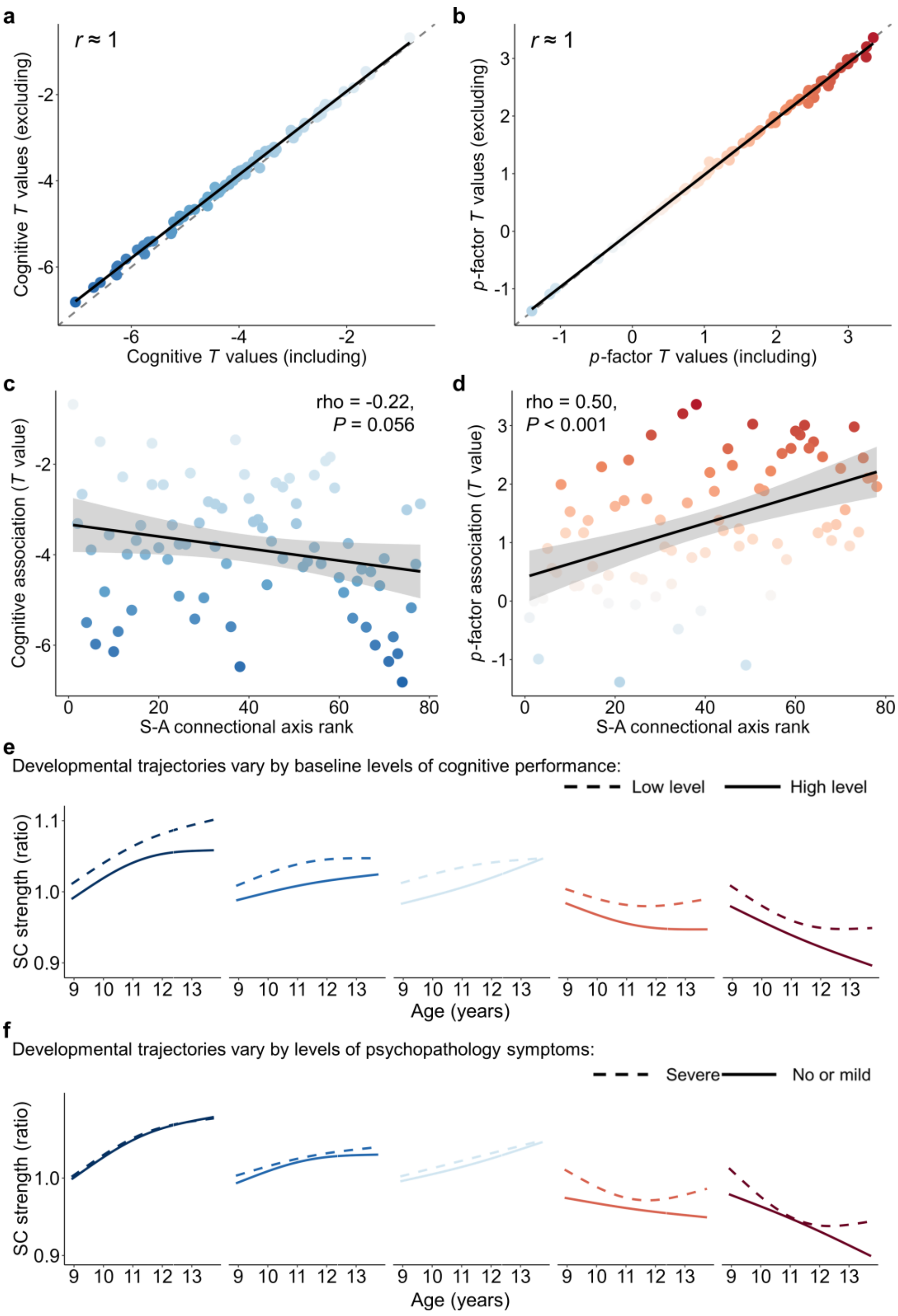

Response Fig. 1. SC–behavior association patterns when behavioral covariates are excluded from data harmonization. a, SC – cognition association patterns obtained when cognitive scores were included versus excluded as ComBat covariates showed a near-perfect correlation (Pearson's $r \approx 1$). **b**, SC – *p*-factor association patterns obtained when *p*-factors were included versus excluded as ComBat covariates showed a near-

perfect correlation (Pearson's $r \approx 1$). The 45° dashed line indicates the line of identity. **c**, Effect sizes (*T* values) of the SC–cognition associations plotted against S-A connectional axis ranks, showing a negative association. **d**, Effect sizes (*T* values) of the SC–*p*-factor associations plotted against S-A connectional axis ranks, showing a positive association. **e**, Developmental trajectories of SC strength are shown for groups with low (10th percentile) and high (90th percentile) cognitive performance across five deciles of the S-A connectional axis. **f**, Developmental trajectories of SC strength are shown for groups with low (10th percentile) and high (90th percentile) *p*-factors across five deciles of the S-A connectional axis. SC: structural connectivity; S-A: sensorimotor-association.

6. Regarding the data harmonization with ComBat, I have a concern that this process may wash out microstructural effects in phenotypic variables that differed between sites. The authors might consider using Neuroharmonize (https://github.com/rpomponio/neuroHarmonize, Pomponio et al., 2020; NeuroImage), which is built off the ComBat algorithm but allows phenotypic differences to be preserved in the harmonization process, especially for developing age youth. Otherwise, is there a way the authors can show harmonization did not affect site differences that might be expected given phenotypic differences?

We appreciate the reviewer's thoughtful suggestion and the recommendation to consider Neuroharmonize. In contrast to traditional ComBat[29], NeuroHarmonize (ComBat-GAM)[33] replaces linear models with generalized additive models (GAMs), allowing nonlinear age effects to be modeled explicitly and thereby better preserved beyond site effects.

In the revised manuscript, we re-harmonized the cross-sectional data using the NeuroHarmonize/ComBat-GAM framework[33]. For the longitudinal ABCD dataset, we applied the NonlinearLongitudinalComBat implementation (https://github.com/nmd1994/NonlinearLongitudinalComBat), which extends the ComBat-GAM framework by incorporating subject-level random intercepts to account for within-subject dependence. Although the devCCNP data in the Chinese Cohort include a longitudinal subset, only a small proportion of participants have repeated measurements (N = 59, 6.8%). Therefore, we did not apply the longitudinal ComBat procedure to the Chinese cohort. In these models, age, sex, and head motion were included as covariates, with age specified as a smooth term. For cognitive and psychopathological analyses, the corresponding cognitive and psychopathological measures were additionally included as covariates to ensure that these phenotypic effects were preserved during harmonization. All results were updated accordingly.

All our findings remained robust when using these alternative harmonization approaches. We have revised the Methods section accordingly and updated the corresponding results throughout the manuscript.

Methods (Page 34, Para 2):

Data from the three datasets were collected across multi-acquisition sites with varied scanners and sequences. To harmonize SC strength across acquisition sites, we applied ComBat-GAM[103] and NonlinearLongitudinalComBat (https://github.com/nmd1994/NonlinearLongitudinalComBat) to the 78 SC connections derived from diffusion MRI data. ComBat-GAM was used for the cross-sectional datasets, whereas NonlinearLongitudinalComBat was applied to the longitudinal ABCD dataset. Both approaches effectively remove site-related batch effects while preserving biological phenotypic effects, particularly nonlinear age-related effects, making them well suited for

developmental datasets. During the harmonization process, covariates included age, sex, and head motion, with age specified as a smooth term. For analyses involving cognitive and psychopathological measures, the corresponding cognitive and psychopathological variables were additionally included as covariates to ensure that these phenotypic effects were preserved during harmonization, following established practice in prior studies[104-106].

Results: All analyses were updated using the suggested approaches, and the results remained stable. We compiled the key results from the primary analyses in **Response Fig. 2**. Please refer to the revised Results section for details.

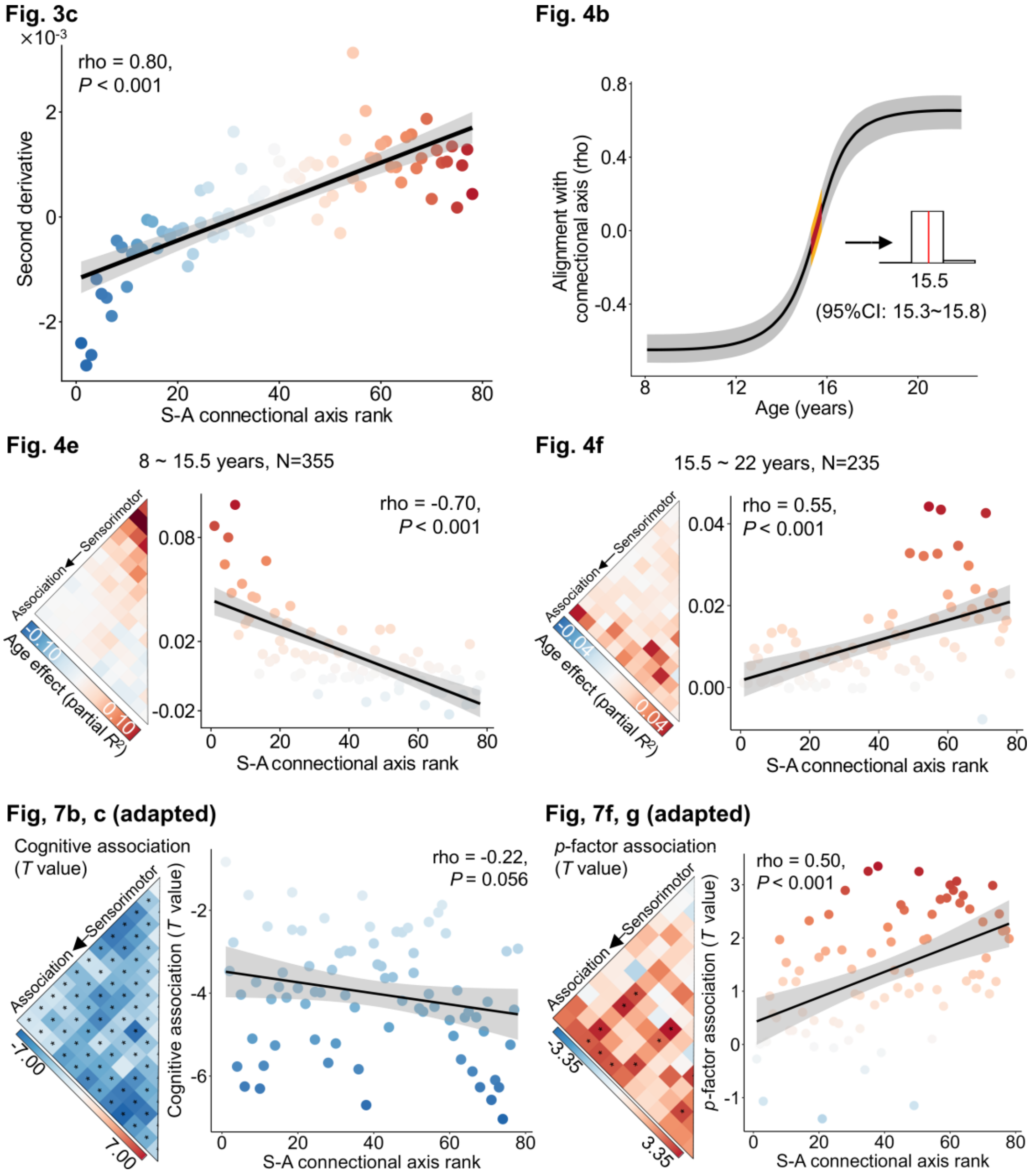

Response Fig. 2. Compilation of primary result panels in the revised manuscript using ComBat-GAM and NonlinearLongitudinalComBat harmonization. Fig. 3c,

Second derivatives of SC trajectories remain highly correlated with S-A connectional axis ranks (rho = 0.80, *P* < 0.001). **Fig. 4b**, Age-resolved alignment between the spatial pattern of SC developmental rates and the S-A connectional axis. Consistent with the original results, alignment increases from negative to positive values and crosses zero around 15.5 years (95% CI: 15.3–15.8). **Fig. 4e**,**f**, Divergent developmental patterns of SC between younger and older youth. **Fig. 7b**,**c**, Fluid composite cognitive scores remain negatively associated with SC strength, and the spatial pattern of SC-cognition associations shows a trend-level alignment with the S-A connectional axis (rho = -0.22, *P* = 0.056). **Fig. 7f**,**g**, SC-*p*-factor associations are positive and show a spatial pattern significantly aligned with the S-A connectional axis (rho = 0.50, *P* < 0.001). S-A: sensorimotor-association; CI: credible interval.

7. The p-factor approach, while increasingly popular, remains controversial. Using parent-reported symptoms in a population-based sample with restricted range may limit validity. Can the authors conduct sensitivity analyses with alternative psychopathology operationalizations that's available with ABCD or other datasets used in this study?

We thank the reviewer for raising this important point and for suggesting sensitivity analyses. We agree that the *p*-factor approach warrants careful validation, particularly when derived from parent-reported symptoms in population-based samples.

First, we note that the *p*-factor is designed to capture continuous liability to psychopathology rather than categorical diagnosis. In the present study, we computed the *p*-factor using the bifactor structure reported by Moore et al. (2020)[34], which was derived and validated within the ABCD dataset. In that study, the bifactor model demonstrated strong psychometric properties, including high construct replicability (H = 0.98) and omega hierarchical (OmegaH = 0.87). In our sample, the model likewise showed good fit (CFI = 0.96, RMSEA = 0.02, SRMR = 0.07) based on the conventional benchmarks[35].

Nevertheless, we acknowledge that *p*-factor estimates may be influenced by sample characteristics. To address this concern, we conducted a sensitivity analysis using an alternative, model-free operationalization of psychopathology available in ABCD: the CBCL Total Problems raw score. As shown in **Fig. S13c**, consistent with the *p*-factor results, the spatial pattern of association effect sizes between CBCL Total Problems and SC strength was significantly aligned with the sensorimotor-association (S-A) connectional axis (rho = 0.51, *P* < 0.001). In addition, the developmental trajectory differences across CBCL Total Problems levels were similar to those observed across *p*-factor levels (**Fig. S13d**). Together, these results indicate that the S-A connectional axis captures systematic spatial variability in SC–psychopathology associations that is robust to the specific operationalization of psychopathology.

We have added this sensitivity analysis and the corresponding results to the revised manuscript:

Methods (Page 26, Para 3):

To evaluate robustness to psychopathology operationalization, we additionally used the CBCL Total Problems raw score as an alternative measure of overall psychiatric symptom burden. The Total Problems score was calculated by summing parent-reported symptom ratings across all CBCL problem items, with higher scores indicating greater overall symptom burden.

Results (Page 17, Para 2):

The alignment of the spatial pattern of SC–psychopathology associations with the S-A connectional axis remained robust when psychopathological burden was indexed using the CBCL Total Problems score (**Fig. S13c**). In addition, stratifying SC trajectories by CBCL Total Problems levels revealed decile-resolved developmental trajectory differences similar to those observed with the *p*-factor (**Fig. S13d**). Together, these results indicate that both the spatial organization of SC–psychopathology associations and the associated developmental patterns are robust to the operationalization of psychopathology.

Supplementary Figure (Page 16):

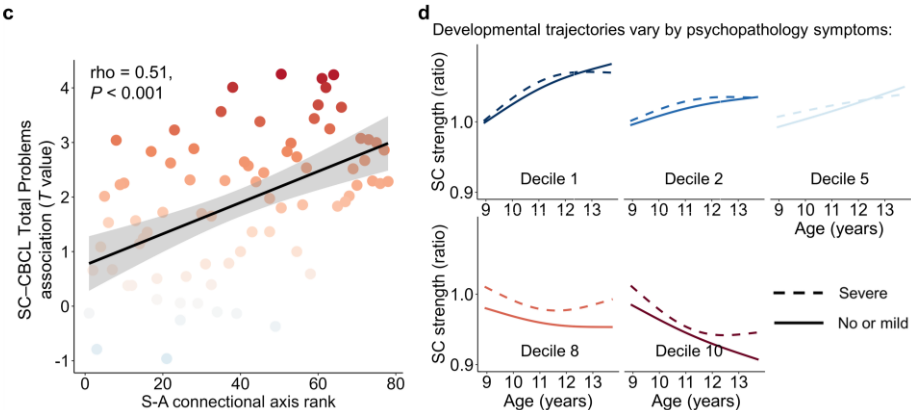

Fig. S13. Sensitivity analyses using alternative measures of cognition and psychopathology. … **c**, The spatial distribution of SC–psychopathology associations was significantly aligned with the S-A connectional axis (rho = 0.51, $P < 0.001$) when psychopathology was indexed using the CBCL Total Problems score. **d**, Developmental trajectories of SC strength are shown at low (10th percentile) and high (90th percentile) CBCL Total Problems scores across five deciles of the S-A connectional axis. CBCL: Child Behavior Checklist; SC: structural connectivity.

8. Despite having longitudinal data from ABCD and partial longitudinal data from devCCNP, the developmental trajectory analyses largely treat data as cross-sectional. The GAMMs with random intercepts account for repeated measures but do not leverage the longitudinal structure to test key claims about within-person developmental change. Statements like "developmental trajectories vary by cognition/psychopathology level" conflate between-person and within-person effects. Can the authors analyze the latent growth curve or multilevel models to explicitly test the within-person change?

We appreciate this important point and agree that our primary GAMM analyses characterize group-level age-associated patterns and do not explicitly disentangle within-person from between-person age effects. Accordingly, moderation by cognition/psychopathology in these models should be interpreted as differences in group-level age associations rather than within-person developmental effects.

In the current study, we included two longitudinal datasets: ABCD (baseline and 2-year follow-up) and devCCNP. However, only 59 participants in devCCNP contributed more than one visit, and substantial variability in baseline age further limited the ability to

estimate within-person age effects in that sample. We therefore focused on the ABCD dataset to directly examine within-person developmental change.

To test within-person effects, we conducted an additional within–between multilevel analysis using linear mixed-effects models (LMM) in the ABCD dataset. Using participants with complete baseline and follow-up data (N = 2,570), we decomposed age into a between-person component (age_bp, baseline age) and a within-person component (age_wp, within-person change in age relative to baseline). Given the two-wave design, we modeled random intercepts only to ensure stable convergence across connections. This design supports estimation of an average within-person linear rate of change but is not suited for detecting nonlinear within-person trajectories, which require more than two time points.

For each of the 78 connections, we fitted the model:

$$\text{SC strength} \sim \text{age\_wp} + \text{age\_bp} + \text{sex} + \text{mean\_fd} + (1 \mid \text{subID}).$$

We extracted the *T* values for age_wp as connection-wise within-person age effects and examined their alignment with the S-A connectional axis. Significant within-person age effects were observed in 70 of the 78 connections. Consistent with the cross-sectional patterns reported in the main analyses (**Fig. 4e**; **Fig. 5g,h**), the spatial pattern of these within-person age effects (**Fig. S9a**) was significantly aligned with the S-A connectional axis (rho = -0.57, $P < 0.001$; **Fig. S9b**). In the revised manuscript, we have incorporated the LMM analysis as a sensitivity analysis.

Methods (Page 29, Para 2):

We further examined within-person developmental effects using ABCD participants with both baseline and two-year follow-up data (N = 2,570). We decomposed age into a between-person component (age_bp, baseline age) and a within-person component (age_wp, the within-person age interval relative to baseline). We then estimated within-person age effects using linear mixed-effects models (equation 2). The *T* values for the age_wp coefficient were extracted as connection-wise estimates of within-person age effects.

$$\text{SC strength} \sim \text{Age\_wp} + \text{Age\_bp} + \text{Sex} + \text{Mean\_FD} + (1 \mid \text{subID}) \quad (2)$$

Results (Page 12, Para 1):

We further examined whether this S-A axis-structured developmental pattern could be detected at the within-person level. Using longitudinal data and linear mixed-effects models, we found that within-person SC age effects were also negatively correlated with S-A axis ranks across connections (rho = -0.57, $P < 0.001$; **Fig. S9a,b**).

Supplementary Materials (Page 12):

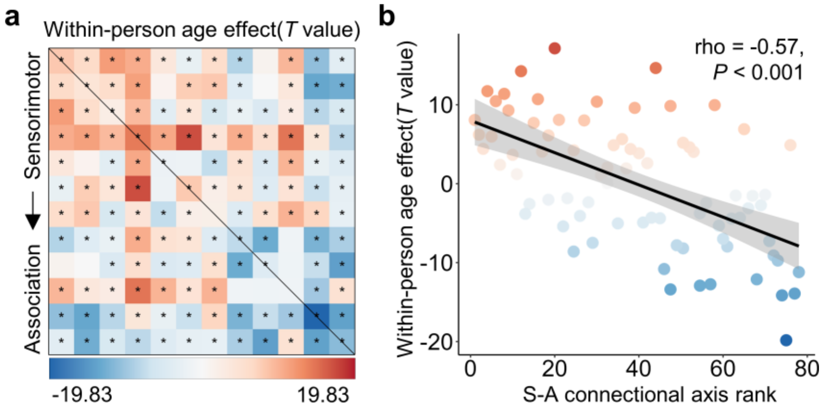

Fig. S9. Within-person age effects in SC strength were associated with the S-A connectional axis ranks. a, Connection-wise within-person age effects estimated from within–between linear mixed-effects models in the ABCD dataset. Values represent *T* statistics for the within-person age term across the 78 system-to-system connections with asterisks indicating significant within-person age effects ($P_{FDR} < 0.05$). **b**, Scatter plot showing the relationship between within-person age effects (*T* values) and S-A connectional axis ranks across the 78 connections. S-A: sensorimotor-association.

We next examined whether within-person age effects differed as a function of cognition and psychopathology by including interactions with the within-person age term (age_wp × cognition; age_wp × *p*-factor). Predicted within-person trajectories were visualized at the 10th and 90th percentiles of cognitive or *p*-factor scores. As our primary analyses, we grouped the 78 connections into deciles and plotted the average trajectories within each decile. As shown in **Response Fig. 3a**, lower cognitive performance was associated with steeper within-person change in lower-ranked connection deciles and slower decline in higher-ranked deciles, broadly consistent with the group-level patterns reported in the main text. However, the magnitude of these within-person moderation effects was modest for both cognition (**Response Fig. 3a**) and psychopathology (**Response Fig. 3b**). These attenuated effects likely reflect the limited ability of two-wave data to capture nonlinear developmental divergence. Specifically, using baseline and 2-year follow-up ABCD assessments, the model captures developmental change over a relatively short two-year window, and with only two time points per participant, longitudinal models are restricted to estimating linear change. Given that our primary analyses indicate difference in nonlinear trajectories by cognitive or psychopathological levels across adolescence, stronger within-person associations between developmental trajectories and behavioral outcomes may emerge over longer follow-up periods or with denser longitudinal sampling, which would better capture nonlinear within-person divergence.

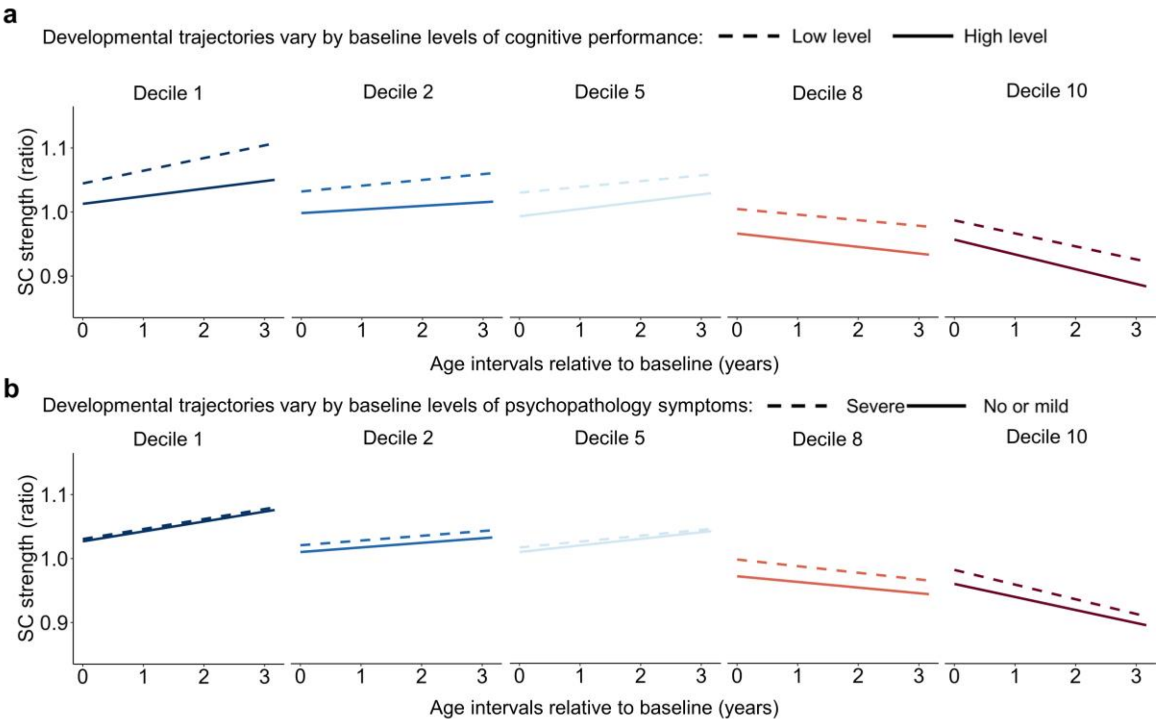

Response Fig. 3. Within-person developmental trajectories across levels of cognition and psychopathology. a, Linear mixed-effects model–predicted trajectories of SC strength are shown for low (10th percentile) and high (90th percentile) cognitive performance across five deciles of the S-A connectional axis. **b**, Linear mixed-effects model–predicted trajectories of SC strength are shown for low (10th percentile) and high (90th percentile) *p*-factors across five deciles of the S-A connectional axis. SC: structural connectivity; S-A: sensorimotor-association.

In the revised manuscript, we clarified that the trajectory differences across cognition and *p*-factor in the primary analyses reflect group-level patterns, and we revised wording that could be misinterpreted as within-person effects.

Abstract (Page 1, Para 1):

Moreover, group-level developmental trajectories of structural connectivity differed by cognitive and psychopathological levels, with psychopathological effects predominantly observed in association connections.

Results (Page 16, Para 2):

Original text:

Youths with lower cognitive scores showed steeper strengthening of sensorimotor connections (deciles 1 and 2) and slower decline in association connections (deciles 8 and 10; **Fig. 6d**).

Revised text:

The developmental trajectories of sensorimotor connections (deciles 1 and 2) were steeper at lower cognitive scores, whereas those of association connections (deciles 8 and 10) showed a slower decline (**Fig. 7d**).

Results (Page 17, Para 2):

Original text:

Youths with higher *p*-factor exhibited earlier inflection and altered strengthening in association-related connections.

Revised text:

Higher *p*-factor was associated with an earlier inflection and altered strengthening in connections near the association end of the S-A axis at the group-level. Notably, the observed differences in developmental trajectories by cognition and psychopathology reflect a combination of between-person and within-person age-related variance. Detecting comparable nonlinear within-person differences will require denser longitudinal sampling.

Finally, we also added a limitation noting that sparse longitudinal sampling restricts inference about within-person effects.

Discussion (Page 23, Para 1):

Additionally, the developmental variability patterns described here primarily reflects group-level effects. Although longitudinal data from the ABCD study were used, participants contributed at most two visits (baseline and two-year follow-up), limiting sensitivity to within-person developmental change and precluding detection of nonlinear transitions in the alignment between the spatial pattern of developmental rates and the S-A axis. Future studies with denser longitudinal sampling and longer follow-up periods may better capture within-person trajectories and their relationships with behavioral outcomes.

9. The “critical transition” at age 15.5 feels over-interpreted. The narrow CI of might suggest potential overfitting reflect the specific parameterization of GAM smoothing rather than a true biological discontinuity. The term “critical” implies a sensitive period with specific biological properties, which is not tested. I wonder if the longitudinal ABCD data show evidence of within-person transitions around this age.

We thank the reviewer for raising this important point. We agree that the term “critical transition” was over-interpreted and that we may have overemphasized a single age estimate (15.5 years), which in fact reflects a broader developmental period. We address each aspect of the comment below.

First, in our primary analyses, the GAM smoothing parameter (k = 3) was selected using AIC with bootstrap validation (**Fig. S14**), in line with prior neurodevelopmental work[20,21,36] using parsimonious smooths to avoid overfitting. To further test whether the estimated transition age depended on the specific smoothing parameterization, we re-fit the HCP-D models with penalized smooths across a range of basis dimensions (k = 4–6). For each k, we recomputed the age at which the spatial correlation between SC change rates and the S-A connectional axis ranks crossed zero. The estimated zero-crossing ages were broadly

consistent, with overlapping 95% credible intervals (CI; *k* = 4: 14.0–15.0 years; *k* = 5: 13.9–15.0 years; *k* = 6: 13.8–14.9 years; **Response Fig. 4**). Across all specifications, we observed the similar pattern: alignment between spatial pattern of developmental rates and the S-A axis shifts from negative to positive around age 15. These results suggest that alignment reversal is not driven by a particular smoothing choice.

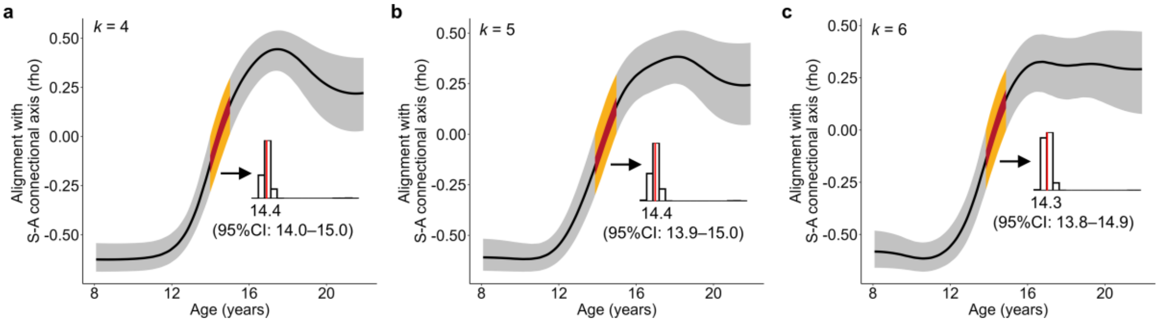

Response Fig. 4. Age-resolved alignment between spatial pattern of SC developmental rates and the S-A connectional axis across different values of *k*. a–c, Developmental models were fitted using a range of basis dimensions (*k* = 4–6) with penalization applied to the smooth term in the HCP-D dataset. Increasing the basis dimension allows greater model flexibility and results in more fluctuating alignment curves. Nevertheless, across all tested values of *k*, the estimated age at which the alignment between SC change rates and the S-A connectional axis ranks crossed zero remained broadly consistent, with overlapping 95% CI (*k* = 4: 14.0–15.0 years; *k* = 5: 13.9–15.0 years; *k* = 6: 13.8–14.9 years). The black line indicates the median correlation across bootstrap samples, the gray band denotes the 95% CI, and the yellow band marks the 95% CI for the age at which the correlation crosses zero. S-A: sensorimotor-association; CI: credible interval.

Second, we acknowledge that the term “critical period” denotes a specific developmental concept referring to temporally restricted windows of heightened neuroplasticity[37]. Because such mechanistic properties were not directly tested in the present study, it is not appropriate to describe the observed alignment transition as a “critical” age. We have therefore removed this terminology and now refer more cautiously to a group-level “inflection” or “transition” in alignment around age 15 in the revised manuscript.

Abstract (Page 1, Para 1):

Original text:

This developmental continuum ranged from early childhood increases in sensorimotor-sensorimotor connectivity strength to late adolescent increases in association-association connectivity strength, with a critical transition around age 15.5.

Revised text:

This developmental continuum ranged from early childhood increases in sensorimotor-sensorimotor connectivity strength to late adolescent increases in association-association connectivity strength, with the transition occurring around age 15.

Results (Page 10, Para 2):

Original text:

The alignment reached zero at 15.5 years (95% CI: 15.3–15.8), indicating a reversal in the spatial organization of connectivity strengthening.

Revised text:

The alignment crossed zero at a median age of 15.5 years (95% CI: 15.3–15.8), indicating a group-level shift in the spatial organization of connectivity strengthening.

Discussion (Page 20, Para 1):

Original text:

The spatial alignment between connectome-wide developmental change rates and the S-A connectional axis evolved during youth, with a critical transition from negative to positive alignment around 15.5 years of age.

Revised text:

The spatial alignment between connectome-wide developmental change rates and the S-A connectional axis evolved during youth, with a transition from negative to positive alignment around 15 years of age.

Finally, regarding the reviewer's question about within-person transitions in the longitudinal ABCD data, in the present study, participants contributed at most two time points (baseline and two-year follow-up). This sampling density permits estimation of linear within-person change but does not support detection of nonlinear within-person inflection points. In addition, the age range covered by the ABCD data in our sample (8.9–13.8 years) is too young to capture a mid-adolescence transition. Accordingly, our findings characterize group-level nonlinear age patterns rather than discrete within-person transitions. We have clarified this limitation and now explicitly note that denser longitudinal sampling a longer follow-up period will be required to test for within-person transitions around mid-adolescence.

Discussion (Page 23, Para 1):

Additionally, the developmental variability patterns described here primarily reflects group-level effects. Although longitudinal data from the ABCD study were used, participants contributed at most two visits (baseline and two-year follow-up), limiting sensitivity to within-person developmental change and precluding detection of nonlinear transitions in the alignment between the spatial pattern of developmental rates and the S-A axis. Future studies with denser longitudinal sampling and longer follow-up periods may better capture within-person trajectories and their relationships with behavioral outcomes.

Minor issues regarding language, typos and awkward wording:

1. P.10 Ln. 16: The alignment was "stale" before ~13 years -> should be "stable"

We sincerely apologize for this oversight. We have corrected and carefully checked the manuscript throughout.

Results (Page 10, Para 2):

The alignment was stable before ~13 years, increased rapidly during mid-adolescence, and stabilized in young adulthood.

2. "pre-defined" "predefined" need to be standardized throughout

Thank you for pointing this out.

Abstract (Page 1, Para 1):

Using diffusion MRI data from three independent developmental cohorts spanning youth, we identified a robust divergence in structural connectivity maturation along a predefined sensorimotor-association (S-A) connectional axis during youth (http://connectcharts.cibr.ac.cn).

3. EFNY's full name needs to be consistent throughout

Thank you.

Acknowledgments (Page 35, Para 1):

We thank the research participants and staff involved in data collection and project executive of the Lifespan Human Connectome Project in Development (HCP-D) study, Adolescent Brain Cognitive Development (ABCD) Study, the Executive Function and Neurodevelopment in Youth (EFNY)……

The Executive Function and Neurodevelopment in Youth (EFNY) data were provided by our lab in CIBR ......

4. hypothesized that the 'spatiotemporal heterogeneity in SC development would have implications in both cognitions' – change to 'hypothesized that the spatiotemporal heterogeneity in SC development would have implications *for both cognition*..'

We appreciate the reviewer's careful inspection.

Introduction (Page 3, Para 1):

As brain development in youth is linked to both higher-order cognition and a variety of mental disorders[3,10,14], we further hypothesized that the spatiotemporal heterogeneity in SC development would have implications for both cognition and psychopathology.

5. Line 19 in Intro- cognition, not 'cognitions'

Thank you for pointing this out.

Introduction (Page 3, Para 1):

……, we further hypothesized that the spatiotemporal heterogeneity in SC development would have implications for both cognition and psychopathology.

Introduction (Page 3, Para 2):

Prior work has shown that increased structural-connectome segregation within association networks is related to better performance in higher-order cognition[3].

Methods (Page 32, Subtitle):

Associations between structural connectivity strength and higher-order cognition

6. Line 19/20 – "hypothesized that large-scale SC strength declined with better cognitive performance.." ; change to 'hypothesized that large-scale SC strength *would * decline with better cognitive performance

Thank you for your correction.

Introduction (Page 3, Para 2):

Therefore, we hypothesized that large-scale SC strength would decline with better cognitive performance, …….

Reviewer #2 (Remarks on code availability):

I can confirm that detailed code is available but have not tried to implement it directly

Thank you for your inspection.

Reviewer #3 (Remarks to the Author):

I co-reviewed this manuscript with one of the reviewers who provided the listed reports. This is part of the Nature Communications initiative to facilitate training in peer review and to provide appropriate recognition for Early Career Researchers who co-review manuscripts.

We thank the reviewer for the constructive comments and hope that the revisions have adequately addressed the points raised.

Reviewer #3 (Remarks on code availability):

Reviewed the general integrity of codes but did not perform a detailed review of specific analysis scripts. Most analyses scripts are available on the github repository with a readme file.

Thank you for reviewing the code availability.

References:

1 Marshall, A. T., Adise, S., Kan, E. C. & Sowell, E. R. Longitudinal Sex-at-Birth and Age Analyses of Cortical Structure in the ABCD Study. *The Journal of neuroscience : the official journal of the Society for Neuroscience* **45** (2025). https://doi.org/10.1523/JNEUROSCI.1091-24.2025

2 Tomasi, D. & Volkow, N. D. Measures of Brain Connectivity and Cognition by Sex in US Children. *JAMA Netw Open* **6**, e230157 (2023). https://doi.org/10.1001/jamanetworkopen.2023.0157

3 Sydnor, V. J. *et al.* Neurodevelopment of the association cortices: Patterns, mechanisms, and implications for psychopathology. *Neuron* **109**, 2820-2846 (2021). https://doi.org/10.1016/j.neuron.2021.06.016

4 Glasser, M. F. & Van Essen, D. C. Mapping human cortical areas in vivo based on myelin content as revealed by T1- and T2-weighted MRI. *The Journal of neuroscience : the official journal of the Society for Neuroscience* **31**, 11597-11616 (2011). https://doi.org/10.1523/JNEUROSCI.2180-11.2011

5 Margulies, D. S. *et al.* Situating the default-mode network along a principal gradient of macroscale cortical organization. *Proc Natl Acad Sci U S A* **113**, 12574-12579 (2016). https://doi.org/10.1073/pnas.1608282113

6 Hill, J. *et al.* Similar patterns of cortical expansion during human development and evolution. *Proc Natl Acad Sci U S A* **107**, 13135-13140 (2010). https://doi.org/10.1073/pnas.1001229107

7 Xu, X. *et al.* Developmental deviations of structural connectivity in youths with ADHD predict symptom and treatment outcomes. *bioRxiv*, 2025.2011.2020.689391 (2025). https://doi.org/10.1101/2025.11.20.689391

8 Oldham, S. & Fornito, A. The development of brain network hubs. *Developmental cognitive neuroscience* **36**, 100607 (2019). https://doi.org/10.1016/j.dcn.2018.12.005

9 Blesa, M. *et al.* Hierarchical Complexity of the Macro-Scale Neonatal Brain. *Cerebral cortex (New York, N.Y. : 1991)* **31**, 2071-2084 (2021). https://doi.org/10.1093/cercor/bhaa345

10 Liang, X. *et al.* Structural connectome architecture shapes the maturation of cortical morphology from childhood to adolescence. *Nat Commun* **15**, 784 (2024). https://doi.org/10.1038/s41467-024-44863-6

11 Ducharme, S. *et al.* Trajectories of cortical thickness maturation in normal brain development--The importance of quality control procedures. *NeuroImage* **125**, 267-279 (2016). https://doi.org/10.1016/j.neuroimage.2015.10.010

12 Bethlehem, R. A. I. *et al.* Brain charts for the human lifespan. *Nature* **604**, 525-533 (2022). https://doi.org/10.1038/s41586-022-04554-y

13 Sun, L. *et al.* Population-specific brain charts reveal Chinese-Western differences in neurodevelopmental trajectories. *bioRxiv*, 2025.2006.2017.659820 (2025). https://doi.org/10.1101/2025.06.17.659820

14 de Faria, O., Jr. *et al.* Periods of synchronized myelin changes shape brain function and plasticity. *Nat Neurosci* **24**, 1508-1521 (2021). https://doi.org/10.1038/s41593-021-00917-2

15 Laumann, T. O. *et al.* Functional System and Areal Organization of a Highly Sampled Individual Human Brain. *Neuron* **87**, 657-670 (2015). https://doi.org/10.1016/j.neuron.2015.06.037

16 Gordon, E. M. *et al.* Precision Functional Mapping of Individual Human Brains. *Neuron* **95**, 791-807 e797 (2017). https://doi.org/10.1016/j.neuron.2017.07.011

17 Du, J. *et al.* Organization of the human cerebral cortex estimated within individuals: networks, global topography, and function. *J Neurophysiol* **131**, 1014-1082 (2024). https://doi.org/10.1152/jn.00308.2023
18 Wang, D. *et al.* Parcellating cortical functional networks in individuals. *Nat Neurosci* **18**, 1853-1860 (2015). https://doi.org/10.1038/nn.4164
19 Cui, Z. *et al.* Individual Variation in Functional Topography of Association Networks in Youth. *Neuron* **106**, 340-353 e348 (2020). https://doi.org/10.1016/j.neuron.2020.01.029
20 Sydnor, V. J. *et al.* Intrinsic activity development unfolds along a sensorimotor-association cortical axis in youth. *Nat Neurosci* (2023). https://doi.org/10.1038/s41593-023-01282-y
21 Luo, A. C. *et al.* Functional connectivity development along the sensorimotor-association axis enhances the cortical hierarchy. *Nature Communications* **15** (2024). https://doi.org/10.1038/s41467-024-47748-w
22 Marek, S. *et al.* Reproducible brain-wide association studies require thousands of individuals. *Nature* **603**, 654-660 (2022). https://doi.org/10.1038/s41586-022-04492-9
23 Liu, S., Abdellaoui, A., Verweij, K. J. H. & van Wingen, G. A. Replicable brain-phenotype associations require large-scale neuroimaging data. *Nat Hum Behav* (2023). https://doi.org/10.1038/s41562-023-01642-5
24 Qu, Y. L. *et al.* Distinct brain network features predict internalizing and externalizing traits in children, adolescents and adults. *Nature Mental Health* **3**, 306-317 (2025). https://doi.org/10.1038/s44220-025-00388-5
25 Pines, A. *et al.* Psychiatric Symptoms, Cognition, and Symptom Severity in Children. *JAMA Psychiatry* (2024). https://doi.org/10.1001/jamapsychiatry.2024.2399
26 Keller, A. S. *et al.* Personalized functional brain network topography is associated with individual differences in youth cognition. *Nat Commun* **14**, 8411 (2023). https://doi.org/10.1038/s41467-023-44087-0
27 Kanhere, A. *et al.* Upper Airway Volume Predicts Brain Structure and Cognition in Adolescents. *Am J Respir Crit Care Med* **211**, 2105-2116 (2025). https://doi.org/10.1164/rccm.202409-1748OC
28 Johnson, W. E., Li, C. & Rabinovic, A. Adjusting batch effects in microarray expression data using empirical Bayes methods. *Biostatistics* **8**, 118-127 (2007). https://doi.org/10.1093/biostatistics/kxj037
29 Fortin, J. P. *et al.* Harmonization of multi-site diffusion tensor imaging data. *NeuroImage* **161**, 149-170 (2017). https://doi.org/10.1016/j.neuroimage.2017.08.047
30 Shafiei, G. *et al.* Reproducible Brain Charts: An open data resource for mapping brain development and its associations with mental health. *Neuron* **113**, 3758-3779 e3756 (2025). https://doi.org/10.1016/j.neuron.2025.08.026
31 Larsen, B. *et al.* Longitudinal Development of Brain Iron Is Linked to Cognition in Youth. *The Journal of neuroscience : the official journal of the Society for Neuroscience* **40**, 1810-1818 (2020). https://doi.org/10.1523/JNEUROSCI.2434-19.2020
32 Hearne, L. J. *et al.* Distinct cognitive and functional connectivity features from healthy cohorts can identify clinical obsessive-compulsive disorder. *Mol Psychiatry* (2025). https://doi.org/10.1038/s41380-025-03416-z
33 Pomponio, R. *et al.* Harmonization of large MRI datasets for the analysis of brain imaging patterns throughout the lifespan. *NeuroImage* **208**, 116450 (2020). https://doi.org/10.1016/j.neuroimage.2019.116450

34 Moore, T. M. *et al.* Criterion validity and relationships between alternative hierarchical dimensional models of general and specific psychopathology. *J Abnorm Psychol* **129**, 677-688 (2020). https://doi.org/10.1037/abn0000601
35 Hu, L. t. & Bentler, P. M. Cutoff criteria for fit indexes in covariance structure analysis: Conventional criteria versus new alternatives. *Structural Equation Modeling: A Multidisciplinary Journal* **6**, 1-55 (1999). https://doi.org/10.1080/10705519909540118
36 Sydnor, V. J. *et al.* Human thalamocortical structural connectivity develops in line with a hierarchical axis of cortical plasticity. *Nature Neuroscience* (2025). https://doi.org/10.1038/s41593-025-01991-6
37 Larsen, B. & Luna, B. Adolescence as a neurobiological critical period for the development of higher-order cognition. *Neuroscience and biobehavioral reviews* **94**, 179-195 (2018). https://doi.org/10.1016/j.neubiorev.2018.09.005

Reviewer #1 (Remarks to the Author):

Thank you for being responsive to my comments. I have no other concerns

We thank the reviewer for reviewing our response and for the valuable comments provided in the previous round of review.

Reviewer #2 (Remarks to the Author):

In this manuscript revision the authors made impressive good faith efforts on most points, including additional data which strengthens the results.
The core developmental finding (S-A axis organization of SC maturation) is robust across datasets, parcellations, and different harmonization approaches. There remain some minor methodological concerns about secondary analysis - for example, there still appears to be some statistical fishing on the cognition correlation, but the effect was always marginal and the authors are now appropriately hedging/tempered the conclusions. In any case, these findings don't fundamentally undermine the main contribution.

We sincerely appreciate the reviewer's recognition of our efforts and the robustness of the main findings. We agree that the SC–cognition associations are modest and should therefore be interpreted with caution. Future advances in white matter tractography, network parcellation, and modeling approaches may help to further clarify these relationships. We also appreciate the reviewer's view that this limitation does not fundamentally undermine the central developmental findings of the manuscript.

Reviewer #2 (Remarks on code availability):

Can confirm that overall the code is available and in a usable state
Thank you for the inspection.

Reviewer #3 (Remarks to the Author):

I co-reviewed this manuscript with one of the reviewers who provided the listed reports. This is part of the Nature Communications initiative to facilitate training in peer review and to provide appropriate recognition for Early Career Researchers who co-review manuscripts.

Thank you for reviewing our manuscript and for your valuable comments.
